# Patterns of Spontaneous Adverse Drug Reaction Reporting in Germany From 2012 to 2021

**DOI:** 10.1002/prp2.70282

**Published:** 2026-06-11

**Authors:** Diana Dubrall, Severin Domgörgen, Patrick Christ, Maike Below, Matthias Schmid, Bernhardt Sachs

**Affiliations:** ^1^ University of Bonn, University Hospital Bonn Institute for Medical Biometry, Informatics and Epidemiology Bonn Germany; ^2^ Research Division Federal Institute for Drugs and Medical Devices (BfArM) Bonn Germany; ^3^ Central Research Institute for Ambulatory Health Care in Germany Berlin Germany; ^4^ Department for Dermatology and Allergy University Hospital RWTH Aachen Aachen Germany

**Keywords:** adverse drug reactions, reporting patterns, reporting rates, spontaneous reporting database, spontaneous reports

## Abstract

The absolute number of spontaneous adverse drug reaction (ADR) reports increased in the past in several national and international ADR databases. However, in most studies drug exposure was not considered. Our study aimed to evaluate the number of ADR reports from Germany in relation to the number of patients with outpatient drug prescriptions stratified by sex, age groups, seriousness, and reporter types (physician versus consumer). Therefore, 407 882 spontaneous ADR reports from Germany, after exclusion of ADR reports related to vaccines, intentional misuses and accidental exposure, submitted between 01.01.2012 and 31.12.2021 were analyzed. The annual numbers of patients with at least one outpatient drug prescription were provided by the Central Research Institute for Ambulatory Health Care in Germany. Reporting rates per year were calculated by dividing the annual number of spontaneous ADR reports by the annual number of patients with at least one outpatient drug prescription. Between 2012 and 2021 (2.4‐fold), and strikingly between 2017 and 2018 (2.2‐fold), the annual reporting rate increased. This increase appears to be primarily driven by non‐serious ADR reports submitted by consumers (32.5‐fold increase), whereas reporting rates for serious ADRs and for reports submitted by physicians remained stable. Furthermore, the rise of non‐serious ADR reports from consumers was particularly apparent for females aged 12–17 and 18–64 years. Overall, the observed increase of non‐serious ADR reports is most likely related to changes in reporting obligations for pharmaceutical companies. Our study suggests that females might report ADRs more frequently.

## 1 Introduction

Adverse drug reactions (ADRs) which occur in everyday clinical practice can be reported spontaneously by health care professionals (HCP, e.g., physicians) or non‐health care professionals (non‐HCP, e.g., consumers or their relatives) to e.g., pharmaceutical companies, professional commissions, or competent authorities [1, 2]. These reports are referred to as spontaneous reports and are stored in spontaneous reporting databases such as EudraVigilance [3], the European ADR database of the European Medicines Agency (EMA). Spontaneous reporting databases are an important tool for analyzing the safety of drug therapy after marketing authorisation in pharmacovigilance practice [4, 5]. One major limitation of spontaneous reporting databases is the unknown extent of underreporting. In 2006, it was estimated that only 5%–10% of all spontaneous ADRs are reported [6]. Additionally, the underreporting may also differ—among other things—between serious and non‐serious ADRs and individual drugs [6, 7].

The absolute number of spontaneous ADR reports increased in the past in several national databases, in EudraVigilance and the global ADR database Vigibase [1, 8, 9, 10]. In EudraVigilance this increase seems to be mainly driven by nonserious ADR reports from non‐HCPs following legislative changes which came into force in 2012, with a translational period until November 2017 [10, 11]. When interpreting the findings of spontaneous reporting databases, drug prescription data should be considered as stated by others [12]. Beyond the number of patients receiving drug prescriptions, external factors such as population size and awareness of ADR reporting systems may also affect the number of reports. Most of the studies referenced above reported absolute numbers or reporting rates per inhabitants and did not account for the number of patients with drug prescriptions. Additionally, reporting rates may differ between high‐, low‐, and middle‐income countries [13]. Overall, the findings from other countries may not be generalisable to Germany due to differences in genetic backgrounds, cultural factors and prescribing behaviors.

Despite the overall increase in ADR reports from non‐HCPs in recent years, less is known about the reporting patterns of non‐HCPs. Adjusting their number of reports for drug‐exposed individuals may help to underscore reporting behaviors of non‐HCPs.

The aim of our analysis was to investigate the temporal patterns of spontaneous ADR reporting between 2012 and 2021 at a population level in Germany. Therefore, reporting rates were calculated by dividing the number of spontaneous ADR reports by the number of patients receiving outpatient prescriptions. Stratified analysis by sex, age groups, seriousness, and reporter types (physicians versus consumer) was performed. Our focus was on providing a more detailed description of reporting patterns of consumer reports, as these reports are becoming increasingly relevant in pharmacovigilance practice and now account for a substantial proportion of all reports. Notably, all analyses were conducted at the population level and therefore describe population‐level trends. Drug‐specific analyses were not the scope of our analysis. Our analysis presents reporting patterns that might be relevant from a pharmacovigilance perspective when interpreting analyses of spontaneous reporting databases.

## 2 Methods

### 2.1 Definitions

ADRs (definition described in literature) are reported by Health Care Professionals (HCPs e.g., physicians, pharmacists) who are obliged by their professional conduct code to report ADRs or by non‐Health Care Professionals (non‐HCP, e.g., consumers) [1, 2]. Following the legal definition, an ADR is classified as serious if the ADR was life‐threatening, led to death, congenital anomalies, hospitalization or prolongation thereof, permanent disabilities or other medically important conditions [2].

### 2.2 EudraVigilance

EudraVigilance, the ADR database of the EMA, contains all spontaneously submitted ADR reports of the member states of the European Economic Area [3]. In EudraVigilance, drugs are coded by the EudraVigilance medicinal product dictionary [14] and ADRs by MedDRA terminology [15].

#### 2.2.1 Identification of Reports

**FIGURE 1:**
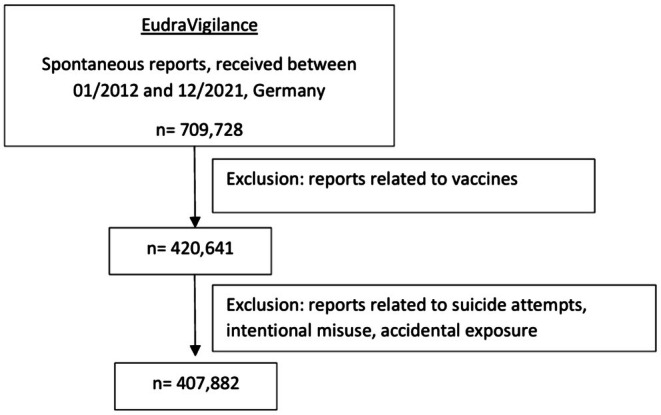
It shows the inclusion and exclusion criteria for the identification of the analysed dataset.

We identified all spontaneous reports from Germany submitted between 01.01.2012 and 31.12.2021 (*n* = 709 728) (Figure 1). ADR reports in which vaccines were reported as suspected/interacting were excluded (*n* = 420 641 reports left). Furthermore, ADR reports describing suicide attempts, intentional misuse and overdoses or accidental exposures were excluded (*n* = 12 739 reports). The final dataset consisted of 407 882 ADR reports.

### 2.3 Outpatient Prescription Data

The annual number of statutory health insured patients in Germany (approximately 80%–90% of the German population) with at least one outpatient prescription (excluding vaccines) dispensed in a German pharmacy between 01.01.2012 and 31.12.2021 was provided by the Central Research Institute for Ambulatory Health Care in Germany according to § 300 SGB V [16]. Patients with only inpatient prescriptions, exclusively private prescriptions, or solely over‐the‐counter drug use without any outpatient prescriptions are not covered.

### 2.4 Performed Analyses

The number of ADR reports per year was determined in total and for certain subgroups. With regard to the subgroups the sex of the patients (female, male, unknown), the respective age groups (0–2 years, 3–11 years, 12–17 years, 18–64 years, 65–85 years, and ≥ 86 years), the seriousness of the ADR reports (yes, no), the ADR reports coded with a hospitalization or prolongation thereof, and the reporter types (physicians, consumers) were considered. For the latter we included only ADR reports explicitly submitted by physicians or consumers to ensure that reporting rates could be clearly attributed to the respective reporter type. Further sub‐analyses were performed within the subgroups, for example, sex within the age groups.

### 2.5 Calculation of Reporting Rates Per Year

Reporting rates per year were calculated by dividing the respective number of reports per year by the respective number of patients with outpatient prescriptions per year. They are presented as the number of reports per 10 000 patients with outpatient prescriptions (= reporting rate).

$$\begin{array}{c} \text{Reporting rate}=\\ \;\frac{\text{Number of reports}\;\mathrm{per}\;\text{year}}{\text{Number of patients with outpatient prescriptions}\;\mathrm{per}\;\text{year}}\times 10,000 \end{array}$$

Notably, one patient may have experienced more than one ADR per year. Thus, in the case of a huge number of patients with more than one ADR report per year, the reporting rate may be overestimated. This also applies to a substantial number of patients with only over‐the‐counter drug use or inpatient prescriptions as these prescriptions are not considered in the denominator. We assume, however, that outpatient drug intake is much more frequent than inpatient drug intake. In addition, most of the patients will also receive outpatient prescriptions after discharge from the hospital. Hence, the non‐consideration of inpatient drug intake should be negligible. Furthermore, previous analyses of our study group showed that most of the ADR reports coded with hospitalization were related to ADRs leading to hospitalization following outpatient prescriptions, whereas only a small proportion were associated with ADRs occurring during inpatient treatment. Thus, the extent of underreporting of ADRs during inpatient treatment may be even greater than underreporting of ADRs following outpatient prescriptions.

Furthermore, for the calculation of the reporting rates, the receive date of the ADR reports was used. Delays in ADR reporting by patients, physicians, and pharmacists together with delays in forwarding ADR reports by pharmaceutical companies (e.g., 15 days for serious ADR reports) may have introduced temporal bias. Thus, some of the ADRs may have occurred in the year before but are assigned to the year thereafter. However, this effect would apply to each year and may diminish any effects.

The ethics committee of the Ärztekammer Nordrhein waived the need for approval since this is not required for retrospective analyses based on pseudonymized spontaneous reports.

## 3 Results

The descriptive analysis of the absolute number of spontaneous ADR reports is presented in Table S1.

### 3.1 Reporting Rates Increased From 2012 to 2021

**FIGURE 2:**
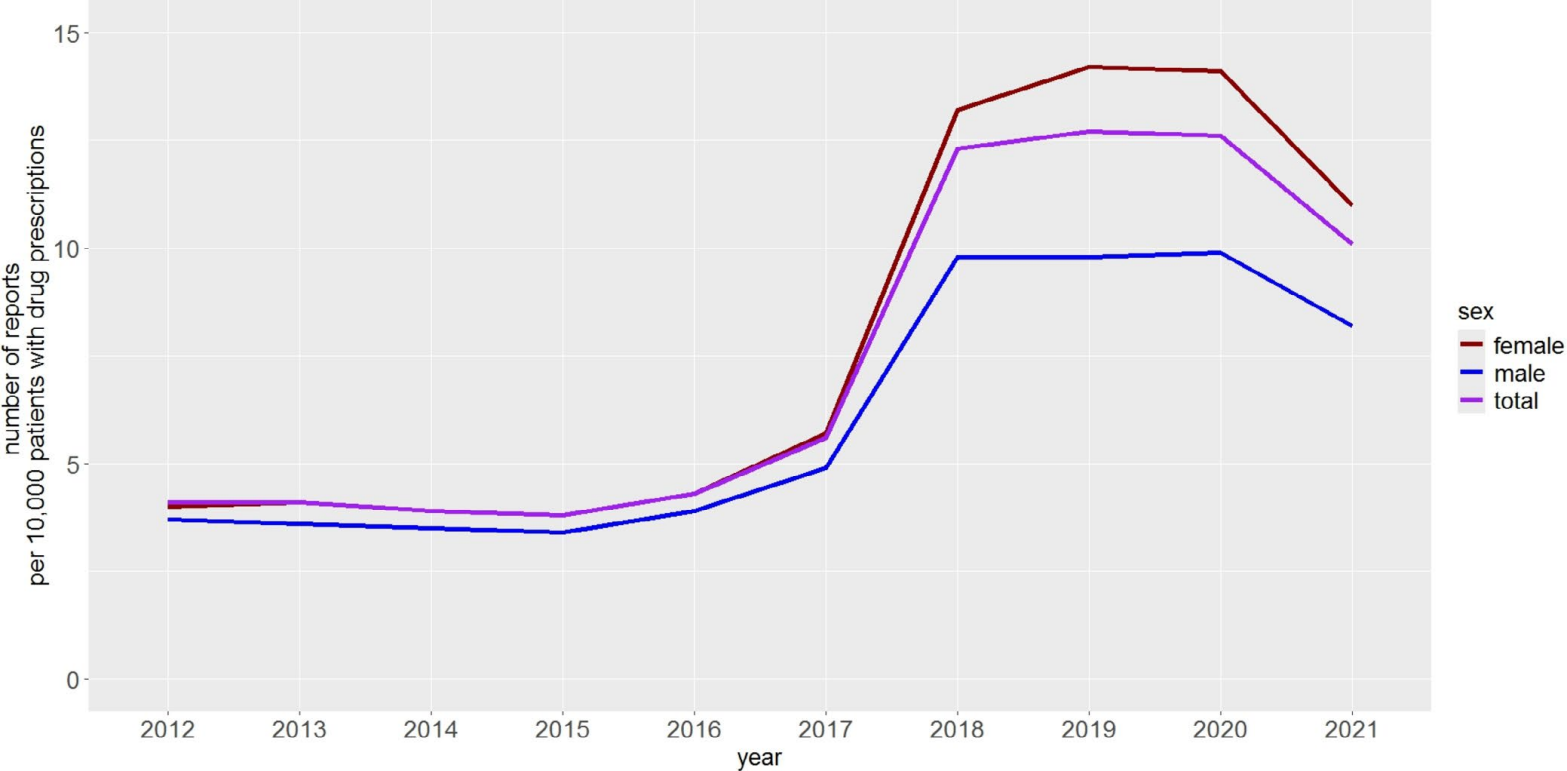
It shows the reporting rates per year from 2012 to 2021 in total and stratified by sex. Note that, ADR reports with unknown sex are also included in the reporting rates of the total ADR reports.

The reporting rates (= number of ADR reports per 10 000 patients with outpatient prescriptions) per year increased from 4.1 reports in 2012 to 12.6 reports in 2020 (3.0‐fold) and decreased to 10.1 reports from 2020 to 2021 (0.8‐fold) (Figure 2). The largest growth (2.2‐fold) was observed between 2017 (5.6 reports) and 2018 (12.3 reports). The overall increase as well as the rise between 2017 and 2018 was higher for ADR reports referring to females (2012–2020: 3.5‐fold, 2017–2018: 2.3‐fold) than for males (2012–2020: 2.7‐fold, 2017–2018: 2.0‐fold). Regarding the absolute number of reports not adjusted for patients with outpatient prescriptions, a lower increase was observed for ADR reports with unknown sex between 2012 and 2020 (1.5‐fold) than for ADR reports concerning females (3.6‐fold) and males (2.8‐fold) (Figure S1).

### 3.2 Reporting Rates Per Year Stratified by Age Groups Increased for Patients Older Than 12 Years

**FIGURE 3:**
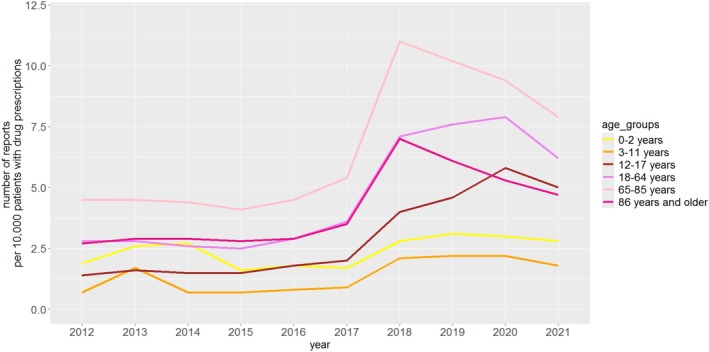
It shows the reporting rates per year from 2012 to 2021 for ADR reports referring to the patients age groups 0–2 years, 3–11 years, 12–17 years, 18–64 years, 65–85 years, and 86 years and older.

Over the entire study period, the highest reporting rates were calculated for patients aged 65–85 years (Figure 3). In addition, this age group showed the largest increase in reporting rates between 2017 and 2018, compared to other age groups. While reporting rates for patients aged 12–17 years, 18–64 years, 65–85 years, and ≥ 86 years clearly increased over time, reporting rates for patients aged 0–2 years and 3–11 years were rather consistent. Stratified by sex, the highest reporting rates were obtained for females aged 18–85 years and males aged ≥ 65 years (Figures S2 and S3). In a direct comparison, the reporting rates for males aged 0–2 years, 3–11 years, 65–85 years, and ≥ 86 years were higher than for females, while the opposite was the case for females aged 12–17 years and 18–64 years compared to their male counterparts (Figures S4–S9). Strikingly, the absolute number of reports referring to patients of unknown age increased clearly in 2018 (2012: 6434 reports, 2020: 28251 reports, increase: 3.4‐fold) (Figure S10). Not only was the absolute number of ADR reports with unknown age higher for females than for males, the increase over time was also greater among females (females: 2012: 3032 reports, 2020: 17253 reports, increase: 5.7‐fold; males: 2012: 2176 reports, 2020: 9340 reports, increase: 4.3‐fold) (Figures S11 and S12).

### 3.3 Reporting Rates for Serious ADRs Per Year Were Consistent

Except for the year 2021, the reporting rates for serious ADRs were rather consistent (range: 2.9–3.4 reports) (Figure S13). In 2021, they slightly declined (2.5 reports). This observation not only applied to the reporting rates, but also to the absolute number of serious ADR reports overall and when analyzed separately for females and males (Figure S14). Compared to that, the number of serious reports with unknown sex declined over time (0.6‐fold). When stratified by age group, the highest reporting rates of serious ADRs were observed among patients aged 65–85 years, followed by those aged ≥ 86 years, whereas the lowest rates were found in patients aged 3–11 years (Figure S15). In view of ADR reports coded with hospitalization or prolongation thereof, increasing reporting rates between 2012 and 2020 were seen for patients aged ≥ 86 (1–8‐fold) and patients aged 12–17 years (2.2‐fold) (Figure S16).

### 3.4 Reporting Rates of Nonserious ADRs Per Year Increased

In contrast to the reporting rates of serious ADRs, the reporting rates of nonserious ADRs increased roughly ten‐fold from 0.9 reports in 2017 to 9.5 reports in 2020 (10.5‐fold) and decreased thereafter to 7.6 reports (2021; 0.8‐fold) (Figure S17). The temporal pattern of the reporting rates of non‐serious ADRs was similar to that observed for ADRs overall (Figure 2). Along with the increasing absolute number of nonserious ADR reports overall and referring to females (2012: 2703 reports, 2020: 33660 reports, increase: 12.5‐fold) and males (2012: 1797 reports, 2020: 17081 reports, increase: 9.5‐fold), this was also seen for patients of unknown sex (2012: 283 reports, 2020: 1513 reports, increase: 5.3‐fold) (Figure S18).

### 3.5 Reporting Rates Per Year of ADRs Reported by Consumers Increased

**FIGURE 4:**
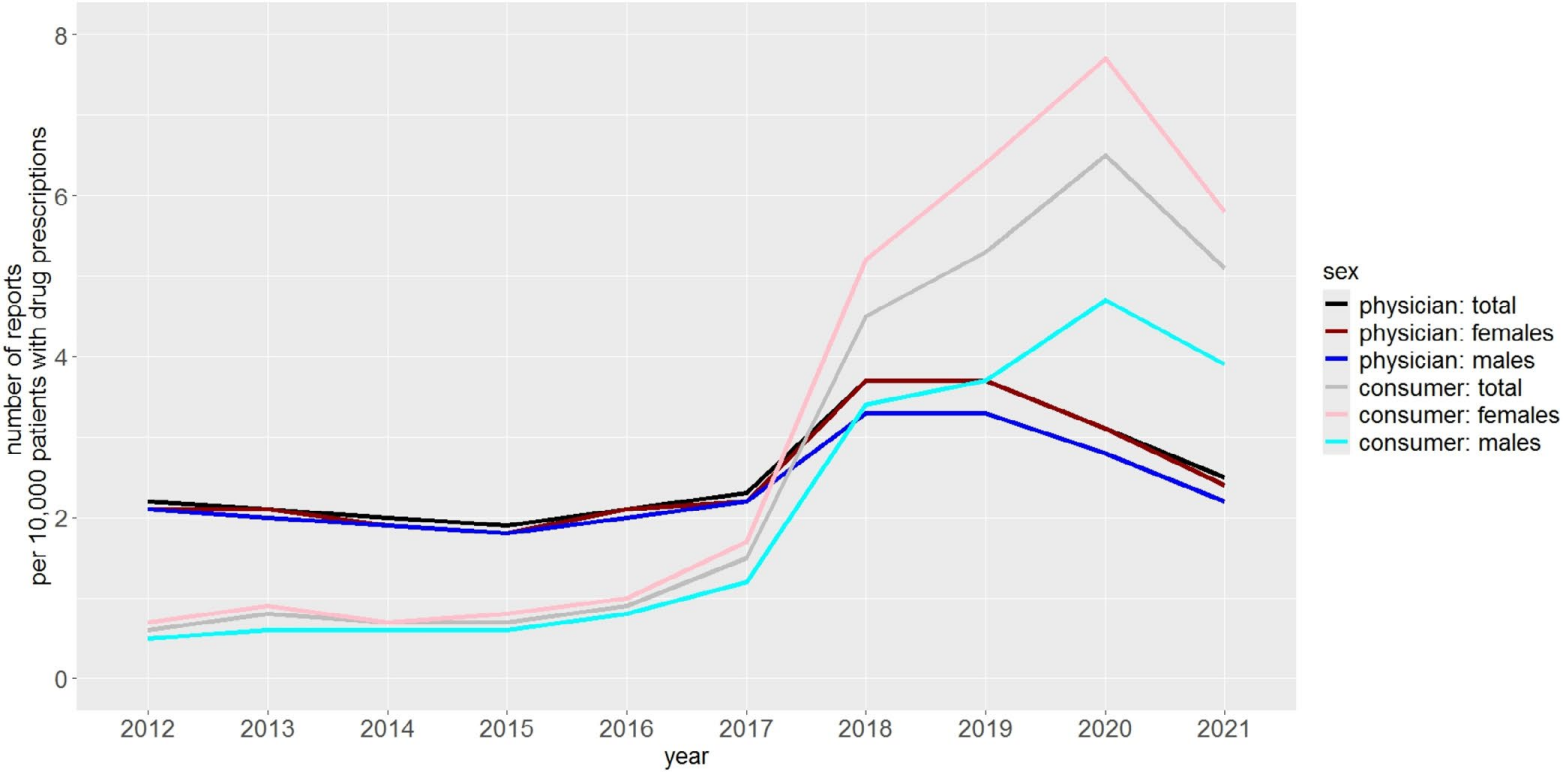
It shows the reporting rates per year from 2012 to 2021 for ADR reports from physicians and consumers in total and referring to females and male. Note that, ADR reports with unknown sex are also included in the reporting rates of the total ADR reports.

Until 2017, the reporting rates from physicians were higher than those from consumers (Figure 4) with similar rates for females and males within each reporter type. After 2018, slightly higher reporting rates for females than for males were observed in ADR reports from physicians. From 2017 onwards, the reporting rates for consumer‐submitted ADRs exceeded those submitted by physicians, particularly for nonserious ADRs concerning females (Figure 4, Figure S19). Stratified by nonserious ADRs, the trend of the reporting rates was similar for both physicians and consumers before 2017, but clearly higher for consumers for both sexes afterwards. In contrast, reporting rates of serious ADRs remained stable over time for both physicians and consumers (Figure S20). Within each reporter group, no relevant differences between females and males were observed. In terms of absolute numbers, a greater increase of ADR reports with patients of unknown sex and unknown age from 2012 to 2020 in ADR reports from consumers (unknown sex: 9.5‐fold, unknown age: 13.9‐fold) compared to those from physicians (unknown sex: 0.8‐fold, unknown age: 1.7‐fold) was noted (Figures S21–S24).

In view of age stratified analyses, the reporting rates for physicians for patients aged 0–2 years, 3–11 years, and 12–17 years were higher than the reporting rates from consumers (Figures S25–S30). For patients aged 18–64 years, 65–85 years, and ≥ 86 years, reporting rates were higher for consumers than physicians, but only after 2017.

## 4 Discussion

This study describes the development of the number of spontaneous ADR reports from Germany in relation to the number of patients with outpatient prescriptions, the so called “reporting rates” over time at a population level. Our findings indicate that the growth in spontaneous ADR reports from Germany is largely attributable to an increase of non‐serious ADR reports from consumers, especially those concerning females aged 12–64 years.

### 4.1 Reporting Rates Serious and Nonserious ADRs From Physicians and Consumers

As in our study, the absolute number of ADR reports increased in the past in several national ADR databases as well as in EudraVigilance [1, 4, 8, 9, 10]. In these studies, drug prescription data were often not considered, but the number of drug prescriptions may affect the number of reports [12, 17]. In our study this increase was not only seen in absolute numbers but also for the calculated reporting rates, particularly between 2017 and 2018. This increase seems to be mainly driven by nonserious ADR reports from consumers. To some extent, this may be related to the amended DIRECTIVE 2010/84/EU, which came into operation in 2012 [11]. In this directive, it was stipulated, among other things, that pharmaceutical companies shall forward all nonserious ADR reports to EudraVigilance. This new requirement came into force with a translational phase until November 2017. Additionally, with the announcement of DIRECTIVE 2010/84/EU all member states of the European Union were requested to encourage ADR reporting by consumers. In Germany, consumers had to be allowed to report suspected ADRs even before 2012 [1]. Additionally, non‐serious ADR reports have already been provided, but to a much lesser extent. Between 1978 and 2016, only 10% of all the spontaneous ADR reports were from consumers and one third were classified as nonserious [1]. An increasing trend of ADR reports from consumers was already observed from 2008 onwards, especially after 2012. Subsequently, between 2018 and 2021 about half of all spontaneous ADR reports from Germany were submitted by consumers and about 87% of them were classified as non‐serious [18]. Besides the legislative changes, a higher awareness of ADRs and how to report them by consumers, the insertion of the call to report ADRs in the package leaflet since 2013 and the opportunity to report ADRs online since 2009 could have impacted the number of ADR reports from consumers in general [1].

Compared with consumer reports, reporting rates for physician‐submitted ADRs increased only slightly from 2017 until 2019, followed by a return to levels observed before 2017. This decrease was particularly apparent for 2021, at the time of the COVID‐19 pandemic and the approval and increased use of the COVID‐19‐vaccines. Other studies have proposed that this decrease in ADR reporting by HCP might be related to limited time for ADR reporting during the pandemic or to a prioritized reporting of ADRs related to COVID‐19‐vaccines over drug‐related ADRs [19]. In addition, patients may also have visited their physicians less frequently during the COVID‐19‐pandemic [20]. Thus, due to the lack of exchange between patients and physicians, physicians may not have been informed about (all) the ADRs experienced by their patients. Even though the reporting rates for consumers were higher than for physicians between 2020 and 2021, a decline was also observed for consumers in 2021. Further studies are needed to investigate reporting rates in the upcoming years.

The extent of underreporting by both HCP and consumers remains a topic of great interest. Importantly, the reporting patterns and obligations changed, and the number of reports increased, which leads to the assumption that most studies examining underreporting years ago are no longer up to date [6, 7, 21, 22, 23]. Additionally, generalizability from other countries to Germany may be limited due to differences in reporting behaviors and knowledge about spontaneous reporting systems. Moreover, most of these studies investigated underreporting by HCP. Compared to that, underreporting by consumers, as a more recent topic, is less well studied. Previous studies concluded that HCP more likely report serious, unknown, and unexpected ADRs to recently authorized drugs [7]. Preferential reporting of serious ADRs by physicians compared to consumers might also be assumed based on our results, although the reporting rates for serious and nonserious ADR reports from physicians were at a similar level after 2017. In other studies, consumers more likely reported serious ADRs along with ADRs affecting their quality of life [24, 25, 26]. The former seems to be contradictory to our findings, as consumers in our study clearly reported more nonserious ADRs. However, this could reflect differences in the definition of seriousness by regulatory authorities, HCP, and consumers as already speculated by others [27]. In our previous study analyzing all spontaneous ADR reports collected between 1978 and 2016 [1], consumers preferentially reported nonspecific ADRs like dizziness, nausea, and headache, which are very likely to be classified as nonserious according to legal criteria, even though they might be severe from a clinical perspective. In a more recent review, lack of knowledge and reservations regarding the spontaneous reporting systems were still the main reasons for underreporting by HCPs [28, 29]. Another review examining interventions to stimulate ADR reporting concluded that educational (e.g., lectures) and engineering strategies (e.g., electronic ADR reporting) were the most successful approaches, at least in the short term [30].

Finally, underreporting may also differ for females, males, and patients of certain age groups. However, less is known about the extent of underreporting for specific patient populations. In summary, further research is needed to investigate the extent of underreporting of ADRs not only for HCPs but also for consumers and specific patient populations and to develop effective interventions to enhance ADR reporting by both HCPs and consumers in the long term.

### 4.2 Reporting Rates of Females, Males, and Certain Age Groups

Especially in ADR reports from consumers, reporting rates were clearly higher for females than for males. This particularly applied to non‐serious ADRs of females aged 12–64 years. Thus, our finding suggests that consumers may more frequently report nonserious ADRs in females aged 12–64 years, as they were more prominently represented in consumer‐submitted reports than in physician‐submitted reports. However, several aspects should be considered when interpreting the calculated reporting rates.

First, a 1.5–1.7‐fold higher overall risk of ADRs in females compared to males has been reported in the literature [17, 31, 32] whereas serious ADRs appeared to be more prevalent among males [17, 33, 34], or at least in older males [33, 35]. Generally, ADRs in females may be detected more often, as females tend to visit physicians more commonly than males [36]. Furthermore, females may take over‐the‐counter drugs more frequently than males; thus, the number of females under risk to potentially develop ADRs might be even higher [37]. Both aspects could lead to an overestimation of the calculated reporting rates for females, as the denominator could not be adjusted for these variables. Especially in younger females, the intake of contraceptives might play a role, a drug class that ranked 10th in our previous analysis of all spontaneous ADR reports collected between 1978 and 2016 [1]. Notably, in Germany, contraceptives can be prescribed at the expense of statutory health insurances until the age of 22 years; afterwards, they are privately prescribed and, thus, not fully covered in our denominator presenting outpatient prescriptions reimbursed by statutory health insurances. Conclusively, this would again lead to an overestimation of the calculated reporting rates for females aged 18–64 years as the number of ADR reports associated with contraceptives is fully included in the numerator. Nevertheless, the more frequent reporting of ADRs by females than by males was already discussed in the literature [34, 38]. Based on our data, we can only evaluate the proportion of females included in our consumer reports, but not whether the respective reporter was a female. It is likely that consumer reports are frequently submitted by the patients themselves, especially among adults; however, this cannot be verified with certainty. Theoretically, the ADR report could have been created by a relative of a different sex. Overall, this finding likely reflects a reporting pattern among consumers. It should not be interpreted as a difference in ADR incidence rates, even though differences in ADR prevalences between the sexes are described in the literature as described above. Further studies are needed to confirm our assumption.

An increase of ADR reports with unknown sex and to a larger extent of ADR reports with unknown age was seen in our study. This was particularly apparent in nonserious ADR reports from consumers. Age and sex are two important variables to evaluate sex‐ and age‐specific ADRs. The causes that led to a less frequent reporting of age and sex in nonserious ADR reports from consumers need to be further investigated. One reason might be the removal of age and sex of the patient to align with data privacy requirements as observed in ADR reports sent via pharmaceutical companies contained in FAERS, the ADR database of the United States of America [39].

### 4.3 Translation Into Pharmacovigilance Practice

To date nonserious ADR reports from consumers account for a large proportion of ADR reports from Germany [16]. Several studies investigating ADR reports from consumers have been carried out in the past [40, 41], but it is unclear whether these still apply as the number of consumer reports has increased remarkably. The in‐depth analysis of ADR reports from consumers is one aim of the project EVAS of our research time which started in May 2024 [42].

Although the quality of ADR reports from consumers has been questioned in previous studies, recent analyses by our research team—based on the presence of specific information in the structured report format—did not show that these reports were of poorer quality than those from HCPs [18]. In this study, ADR reports submitted by consumers and physicians demonstrated a similar degree of completeness regarding the information provided. Moreover, other factors such as older patients taking more than one drug had a greater impact on the completeness of information provided independent of the reporting source [43].

Whether the higher number of non‐serious ADR reports from consumers, especially of those from females, will impact signal detection or increase the signal‐to‐noise ratio should be the subject of further studies. One study performed in the second quarter of 2019 concluded that the higher number of non‐serious ADR reports in EudraVigilance did not affect the efficiency of signal detection [10]. However, this study included all ADR reports transmitted to EudraVigilance until June 2019, but the number of reports from consumers remained at a high level thereafter. Additionally, in Germany, the proportion of ADR reports from consumers might be higher than in other countries, limiting the transferability of the results. The authors already stated that their impact on signal detection needs to be further investigated in the future.

In general, it should be recommended—whenever possible—to consider spontaneous ADR reports in relation to the number of drug prescriptions. Nevertheless, our study may help researchers without access to detailed drug prescription data to interpret analysis of spontaneous reporting databases.

### 4.4 Strengths and Limitations

One of the major strengths of our analysis is the consideration of the number of ADR reports in context with outpatient prescriptions. This allows us to make further assumptions regarding the temporal development of the number of ADR reports from Germany in the study period excluding the influence of the number of individuals receiving outpatient prescriptions, which differ per year, sex, and age group.

The unknown extent of underreporting is one of the major limitations of our analysis and current estimates of the extent of underreporting by HCP, consumers, females, males, and certain age groups in Germany are not available as already discussed above. Another limitation lies in the method of calculating the reporting rates. First, our denominator only includes patients with at least one outpatient prescription per year. The number of patients with only inpatient prescriptions, and the number of patients taking only over‐the‐counter drugs without any outpatient prescriptions could not be considered as these data are not available for Germany. Additionally, the fact that a drug has been prescribed does not necessarily mean that it has been taken, as no information on patient adherence to prescribed medication was available. Furthermore, neither does our denominator account for the duration of drug exposure nor for polypharmacy and co‐medication. Conclusively, we could not adjust our reporting rates for all these factors. Second, the numerator may include one patient multiple times in case of more than one ADR reported per year, as we were not able to identify such reports and exclude them from further analysis. This again could influence the calculated reporting rates. Moreover, our analysis only shows patterns of ADR reporting at a population level, but reporting rates are different for individual drugs and ADRs. However, that was not the aim of our study. This could be investigated in future studies.

Our findings are presented as reporting rates. They do not represent incidence rates as incidences cannot be calculated based on spontaneous reporting analyses. Notably, the reporting rates may also be influenced by stimulated reporting, for example, due to media attention or dear doctor letters. Finally, this analysis includes reports of suspected ADRs, the causal relationship between the reported ADR and the suspected drug(s) was not evaluated.

## 5 Conclusion

Our analysis shows a huge increase of the number of ADR reports from Germany between 2012 and 2021 (2.4‐fold) largely induced by an increase of non‐serious ADR reports from consumers (32.5‐fold), which is most likely related to changes of reporting obligations for pharmaceutical companies. Females were more prevalent in the ADR reports from consumers, which could indicate that they report ADRs more frequently. However, this finding has to be confirmed in further studies. The extent of underreporting and the impact of the increasing numbers of consumer reports on signal detection should be further investigated in future studies.

## Author Contributions

**Diana Dubrall:** conceptualization, data curation, formal analysis, investigation, methodology, visualization, writing – original draft. **Severin Domgörgen:** conceptualization, writing – review and editing. **Patrick Christ:** conceptualization, writing – review and editing. **Maike Below:** resources, writing – review and editing. **Matthias Schmid:** methodology, supervision, writing – review and editing. **Bernhardt Sachs:** conceptualization, supervision, writing – review and editing.

## Funding

EVAS is funded by the German Federal Institute for Drugs and Medical Devices' (BfArM) own resources and by the Institute for Medical Biometry, Informatics, and Epidemiology (IMBIE), University Hospital of Bonn, Germany (V‐2023.3/68502/2024‐2028).

## Disclosure

The information and views set out in this manuscript are those of the authors and do not necessarily reflect the official opinion of the Federal Institute for Drugs and Medical Devices.

## Ethics Statement

The ethics committee of the Ärztekammer Nordrhein waived the need for approval since this is not required for retrospective analyses based on pseudonymized spontaneous reports. No surveys or examinations were carried out on patients. The analyzed data is routinely collected and stored in the adverse drug reaction database EudraVigilance from the European Medicines Agency (EMA). Among others, pharmaceutical companies, physicians, pharmacists, and patients report these ADRs (information concerning the reporting channels see Dubrall et al., 2018 (25)). Further information concerning the processing of personal data in the context of the operation of EudraVigilance Human can be found in the European Medicines Agency's Data Protection Notice for EudraVigilance Human (https://www.ema.europa.eu/en/documents/other/european‐medicines‐agencys‐data‐protection‐notice‐eudravigilance‐human‐ev_en.pdf). The Federal Institute for Drugs and Medical Devices (BfArM) as a national competent authority is granted the highest level of access to EudraVigilance since one of the core duties of the BfArM is to analyze EudraVigilance data in order to fulfill its pharmacovigilance obligations.

## Consent

The authors have nothing to report.

## Conflicts of Interest

D.D. and M.S. are supported by the EVAS project, which is founded by the Federal Institute for Drugs and Medical Devices and the Institute for Medical Biometry, Informatics and Epidemiology at the University Hospital Bonn. All other authors have no conflicts of interest to declare.

## Supporting information


**Table S1:** Descriptive analysis of the dataset (*n* = 407 882).
**Figure S1:** Number of reports referring to females, males and unknown sex.
**Figure S2:** Reporting rates of ADR reports referring to females of specific age groups per 10 000 patients with outpatient drug prescriptions per year.
**Figure S3:** Reporting rates of ADR reports referring to males of specific age groups per 10 000 patients with outpatient drug prescriptions per year.
**Figure S4:** Reporting rates of ADR reports referring to patients aged 0–2 years per 10 000 patients with outpatient drug prescriptions per year.
**Figure S5:** Reporting rates of ADR reports referring to patients aged 3–11 years per 10 000 patients with outpatient drug prescriptions per year.
**Figure S6:** Reporting rates of ADR reports referring to patients aged 12–17 years per 10 000 patients with outpatient drug prescriptions per year.
**Figure S7:** Reporting rates of ADR reports referring to patients aged 18–64 years per 10 000 patients with outpatient drug prescriptions per year.
**Figure S8:** Reporting rates of ADR reports referring to patients aged 65–85 years per 10 000 patients with outpatient drug prescriptions per year.
**Figure S9:** Reporting rates of ADR reports referring to patients aged ≥ 86 years per 10 000 patients with outpatient drug prescriptions per year.
**Figure S10:** Number of ADR reports referring to patients of specific age groups per year.
**Figure S11:** Number of ADR reports referring to females of specific age groups per year.
**Figure S12:** Number of ADR reports referring to males of specific age groups per year.
**Figure S13:** Reporting rates of serious ADR reports in total and referring to females and males per 10 000 patients with outpatient drug prescriptions per year.
**Figure S14:** Number of serious ADR reports referring to females, males and unknown sex per year.
**Figure S15:** Reporting rates of serious ADR reports for specific age groups per 10 000 patients with outpatient drug prescriptions per year.
**Figure S16:** Reporting rates of ADR reports coded with hospitalization or prolongation thereof for specific age groups per 10 000 patients with outpatient drug prescriptions per year.
**Figure S17:** Reporting rates of non‐serious ADR reports in total and referring to females and males per 10 000 patients with outpatient drug prescriptions per year.
**Figure S18:** Number of non‐serious ADR reports referring to females, males and unknown sex per 10 000 patients with outpatient drug prescriptions per year.
**Figure S19:** Reporting rates of non‐serious ADR reports from physicians and consumers referring to females and males per 10 000 patients with outpatient drug prescriptions per year.
**Figure S20:** Reporting rates of serious ADR reports from physicians and consumers referring to females and males per 10 000 patients with outpatient drug prescriptions per year.
**Figure S21:** Number of ADR reports from consumers.
**Figure S22:** Number of ADR reports from physicians.
**Figure S23:** Number of ADR reports from consumers per age group.
**Figure S24:** Number of ADR reports from physicians per age group.
**Figure S25:** Reporting rates of ADR reports from physicians and consumers referring to patients aged 0–2 years per 10 000 patients with outpatient drug prescriptions per year.
**Figure S26:** Reporting rates of ADR reports from physicians and consumers referring to patients aged 3–11 years per 10 000 patients with outpatient drug prescriptions per year.
**Figure S27:** Reporting rates of ADR reports from physicians and consumers referring to patients aged 12–17 years per 10 000 patients with outpatient drug prescriptions per year.
**Figure S28:** Reporting rates of ADR reports from physicians and consumers referring to patients aged 18–64 years per 10 000 patients with outpatient drug prescriptions per year.
**Figure S29:** Reporting rates of ADR reports from physicians and consumers referring to patients aged 65–85 years per 10 000 patients with outpatient drug prescriptions per year.
**Figure S30:** Reporting rates of ADR reports from physicians and consumers referring to patients aged ≥ 86 years per 10 000 patients with outpatient drug prescriptions per year.

## Data Availability

The pseudonymised ADR reports from EudraVigilance are not publicly accessible due to data protection requirements. Distinct levels of access are provided for various stakeholders (https://www.ema.europa.eu/en/human‐regulatory/research‐development/pharmacovigilance/eudravigilance/access‐eudravigilance‐data). Being one of the competent authorities in Germany, the highest level of access is granted to the Federal Institute for Drugs and Medical Devices (BfArM). Nevertheless, even with the lowest access level, researchers can perform the same analysis in EudraVigilance (EV) with aggregated data (public access: www.adrreports.eu/en/index.html). For further information regarding processing personal data in the context of the operation of EudraVigilance Human, we refer to the European Medicines Agency's Data Protection Notice for EudraVigilance Human.

## References

[1] D. Dubrall , M. Schmid , E. Alešik , N. Paeschke , J. Stingl , and B. Sachs , “Frequent Adverse Drug Reactions, and Medication Groups Under Suspicion,” Deutsches Ärzteblatt International 115, no. 23 (2018): 393–400. 29960607 10.3238/arztebl.2018.0393 PMC6041966

[2] European Medicines Agency , “Guideline on Good Pharmacovigilance Practices (GVP) Module VI – Collection, Management and Submission of Reports of Suspected Adverse Reactions to Medicinal Products (Rev 2),” https://www.ema.europa.eu/en/documents/regulatory‐procedural‐guideline/guideline‐good‐pharmacovigilance‐practices‐gvp‐module‐vi‐collection‐management‐and‐submission‐reports‐suspected‐adverse‐reactions‐medicinal‐products‐rev‐2_en.pdf.

[3] European Medicines Agency , “EudraVigilance,” https://www.ema.europa.eu/en/human‐regulatory‐overview/research‐development/pharmacovigilance‐research‐development/eudravigilance.

[4] European Medicines Agency , “2022 Annual Report on EudraVigilance for the European Parliament, the Council and the Commission Reporting Period: 1 January to 31 December 2022,” https://www.ema.europa.eu/en/documents/report/2022‐annual‐reporteudravigilance‐european‐parliament‐council‐and‐commission_en.pdf.

[5] N. Moore , D. Berdaï , P. Blin , and C. Droz , “Pharmacovigilance – The Next Chapter,” Thérapie 74 (2019): 557–567. 31623850 10.1016/j.therap.2019.09.004

[6] L. Hazell and S. A. W. Shakir , “Under‐Reporting of Adverse Drug Reactions: A Systematic Review,” Drug Safety 29, no. 5 (2006): 385–396. 16689555 10.2165/00002018-200629050-00003

[7] J. Hasford , M. Goettler , K. H. Munter , and B. Müller‐Oerlinghausen , “Physicians' Knowledge and Attitudes Regarding the Spontaneous Reporting System for Adverse Drug Reactions,” Journal of Clinical Epidemiology 55, no. 9 (2002): 945–950. 12393084 10.1016/s0895-4356(02)00450-x

[8] G. Ozcan , E. Aykac , Y. Kasap , N. T. Nemutlu , E. Sen , and N. D. Aydinkarahaliloglu , “Adverse Drug Reaction Reporting Pattern in Turkey: Analysis of the National Database in the Context of the First Pharmacovigilance Legislation,” Drugs ‐ Real World Outcome 3, no. 1 (2016): 33–43. 10.1007/s40801-015-0054-1 PMC4819489 27747800

[9] F. Thiessard , E. Roux , G. Miremont‐Salamé , et al., “Trends in Spontaneous Adverse Drug Reaction Reports to the French Pharmacovigilance System (1986–2001),” Drug Safety 28, no. 8 (2005): 731–740. 16048358 10.2165/00002018-200528080-00007

[10] G. Candore , S. Monzon , J. Slattery , et al., “The Impact of Mandatory Reporting of Non‐Serious Safety Reports to EudraVigilance on the Detection of Adverse Reactions,” Drug Safety 45 (2022): 83–95. 34881404 10.1007/s40264-021-01137-0 PMC8763735

[11] “DIRECTIVE 2001/83/EC OF THE EUROPEAN PARLIAMENT AND OF THE COUNCIL of 6 November 2001 on the Community Code Relating to Medicinal Products for Human Use,” https://eur‐lex.europa.eu/LexUriServ/LexUriServ.do?uri=CONSLEG:2001L0083:20121116:EN:PDF.

[12] K. Svendsen , K. H. Halvorsen , S. Vorren , H. Samdal , and B. Garcia , “Adverse Drug Reaction Reporting: How Can Drug Consumption Information Add to Analyses Using Spontaneous Reports?,” European Journal of Clinical Pharmacology 74, no. 4 (2018): 497–504. 29255992 10.1007/s00228-017-2396-y

[13] L. Aagaard , J. Strandell , L. Melskens , P. S. Petersen , and E. Holme Hansen , “Global Patterns of Adverse Drug Reactions Over a Decade: Analyses of Spontaneous Reports to VigiBase,” Drug Safety 35, no. 12 (2012): 1171–1182. 23072620 10.1007/BF03262002

[14] European Medicines Agency , “Extended EudraVigilance Medicinal Product Dictionary (XEVMPD) Training,” https://www.ema.europa.eu/en/human‐regulatory/post‐authorisation/data‐medicines‐iso‐idmp‐standards/extended‐eudravigilance‐medicinal‐product‐dictionary‐xevmpd‐training.

[15] “Medical Dictionary for Regulatory Activities (MedDRA),” https://www.meddra.org/. 10.2165/00002018-199920020-00002 10082069

[16] “Central Research Institute for Ambulatory Health Care in Germany,” https://www.zi.de/.

[17] P. Christ , D. Dubrall , K. S. Just , et al., “Identification and Comparison of Sex‐Specific Serious Adverse Drug Reactions in Spontaneous Reports and Systematically Collected Reports (ADRED),” British Journal of Clinical Pharmacology 90, no. 3 (2024): 776–792. 37897066 10.1111/bcp.15941

[18] P. Christ , D. Dubrall , M. Schmid , and B. Sachs , “Comparative Analysis of Information Provided in German Adverse Drug Reaction Reports Sent by Physicians, Pharmacists and Consumers,” Drug Safety 46, no. 12 (2023): 1363–1379. 37987966 10.1007/s40264-023-01355-8 PMC10684666

[19] S. De Germay , A. Singier , F. Salvo , A. Pariente , and French Pharmacovigilance Network , “Impact of Covid‐19 Vaccination on Spontaneous Pharmacovigilance Reporting in France,” Drug Safety 46, no. 12 (2023): 1381–1389. 37926785 10.1007/s40264-023-01359-4

[20] L. A. Kapsner , M. O. Kampf , S. A. Seuchter , et al., “Reduced Rate of Inpatient Hospital Admissions in 18 German University Hospitals During the COVID‐19 Lockdown,” Frontiers in Public Health 8 (2021): 594117. 33520914 10.3389/fpubh.2020.594117 PMC7838458

[21] A. Sandberg , V. Salminen , S. Heinonen , and M. Sivén , “Under‐Reporting of Adverse Drug Reactions in Finland and Healthcare Professionals' Perspectives on How to Improve Reporting,” Healthcare 10, no. 6 (2022): 1015. 35742066 10.3390/healthcare10061015 PMC9222550

[22] M. Khalili , B. Mesgarpour , H. Sharifi , A. Golozar , and A. A. Haghdoost , “Estimation of Adverse Drug Reaction Reporting in Iran: Correction for Underreporting,” Pharmacoepidemiology and Drug Safety 30, no. 8 (2021): 985–1142. 33772938 10.1002/pds.5235

[23] Y. Moride , F. Haramburu , A. Requerjo , and B. Begaud , “Under‐Reporting of Adverse Drug Reactions in General Practice,” British Journal of Clinical Pharmacology 43 (1997): 177–181. 9131950 10.1046/j.1365-2125.1997.05417.x PMC2042725

[24] S. T. De Vries , P. Denig , A. Andrić , et al., “PGM‐IMI Web‐RADR Work Package 3b Consortium and SCOPE Joint Action Work Package 4. Motives to Report Adverse Drug Reactions to the National Agency: A Survey Study Among Healthcare Professionals and Patients in Croatia, The Netherlands, and the UK,” Drug Safety 44, no. 10 (2021): 1073–1083. 34368940 10.1007/s40264-021-01098-4 PMC8473351

[25] C. Matos , F. van Hunsel , and J. Joaquim , “Are Consumers Ready to Take Part in the Pharmacovigilance System? A Portuguese Preliminary Study Concerning ADR Reporting,” European Journal of Clinical Pharmacology 71, no. 7 (2015): 883–890. 26004569 10.1007/s00228-015-1867-2

[26] R. Al Dweik , S. Yaya , D. Stacey , and D. Kohen , “Patients' Experiences on Adverse Drug Reactions Reporting: A Qualitative Study,” European Journal of Clinical Pharmacology 76, no. 12 (2020): 1723–1730. 32661571 10.1007/s00228-020-02958-1

[27] N. D. Aydınkarahaliloğlu , E. Aykaç , Ö. Atalan , N. Demir , and M. Hayran , “Spontaneous Reporting of Adverse Drug Reactions by Consumers in Comparison With Healthcare Professionals in Turkey From 2014 to 2016,” Pharmaceutical Medicine 32 (2018): 353–364.

[28] P. García‐Abeijon , C. Costa , M. Taracido , M. T. Herdeiro , C. Torre , and A. Figueiras , “Factors Associated With Underreporting of Adverse Drug Reactions by Health Care Professionals: A Systematic Review Update,” Drug Safety 46, no. 7 (2023): 625–636. 37277678 10.1007/s40264-023-01302-7 PMC10279571

[29] F. R. Varallo , S. O. Guimarães , S. A. Abjaude , and P. C. Mastroianni , “Causes del subregisto de los eventos adversos de medicamentos por los profesionales de la salud: revision sistemática. [Causes for the Underreporting of Adverse Drug Events by Health Professionals: A Systematic Review],” Revista da Escola de Enfermagem da USP 48, no. 4 (2014): 739–747. 10.1590/s0080-623420140000400023 25338257

[30] P. A. Routledge and R. Bracchi , “Improving the Spontaneous Reporting of Suspected Adverse Drug Reactions: An Overview of Systematic Reviews,” British Journal of Clinical Pharmacology 89, no. 8 (2023): 2377–2385. 37194555 10.1111/bcp.15791

[31] R. M. Martin , P. N. Biswas , S. N. Freemantle , G. L. Pearce , and R. D. Mann , “Age and Sex Distribution of Suspected Adverse Drug Reactions to Newly Marketed Drugs in General Practice in England: Analysis of 48 Cohort Studies,” British Journal of Clinical Pharmacology 46, no. 5 (1998): 505–511. 9833605 10.1046/j.1365-2125.1998.00817.x PMC1873702

[32] Y. Zopf , C. Rabe , and A. Neubert , “Women Encounter ADRs More Often Than Do Men,” European Journal of Clinical Pharmacology 64, no. 10 (2008): 999–1004. 18604529 10.1007/s00228-008-0494-6

[33] L. Holm , E. Ekman , and K. Jorsäter Blomgren , “Influence of Age, Sex and Seriousness on Reporting of Adverse Drug Reactions in Sweden,” Pharmacoepidemiology and Drug Safety 26, no. 3 (2017): 335–343. 28071845 10.1002/pds.4155

[34] S. Watson , O. Caster , P. A. Rochon , and H. den Ruijter , “Reported Adverse Drug Reactions in Women and Men: Aggregated Evidence From Globally Collected Individual Case Reports During Half a Century,” EClinicalMedicine 17 (2019): 100188. 31891132 10.1016/j.eclinm.2019.10.001 PMC6933269

[35] D. Dubrall , K. S. Just , M. Schmid , J. C. Stingl , and B. Sachs , “Adverse Drug Reactions in Older Adults: A Retrospective Comparative Analysis of Spontaneous Reports to the German Federal Institute for Drugs and Medical Devices,” BMC Pharmacology and Toxicology 21, no. 1 (2020): 25. 32293547 10.1186/s40360-020-0392-9 PMC7092423

[36] Robert Koch‐Institut , “Public Dashboard,” https://public.tableau.com/app/profile/robert.koch.institut/viz/Gesundheit_in_Deutschland_aktuell/GEDA_20192020‐EHIS.

[37] E. Barrenberg , H. Knopf , and E. Garbe , “Over‐The‐Counter (OTC) Drug Consumption Among Adults Living in Germany: Results From the German Health Interview and Examination Survey for Adults 2008^−^2011 (DEGS1),” Pharmacy (Basel) 6, no. 2 (2018): 52. 29880765 10.3390/pharmacy6020052 PMC6024976

[38] M. Rademaker , “Do Women Have More Adverse Drug Reactions?,” American Journal of Clinical Dermatology 2, no. 6 (2001): 349–351, 10.2165/00128071-200102060-00001. 11770389

[39] P. Pham , C. Cheng , E. Wu , et al., “Leveraging Case Narratives to Enhance Patient Age Ascertainment From Adverse Event Reports,” Pharmaceutical Medicine 35, no. 5 (2021): 307–316. 34476768 10.1007/s40290-021-00398-5 PMC9136956

[40] L. Aagaard , L. H. Nielsen , and E. H. Hansen , “Consumer Reporting of Adverse Drug Reactions: A Retrospective Analysis of the Danish Adverse Drug Reaction Database From 2004 to 2006,” Drug Safety 32, no. 11 (2009): 1067–1074. 19810778 10.2165/11316680-000000000-00000

[41] A. J. Avery , C. Anderson , C. M. Bond , et al., “Evaluation of Patient Reporting of Adverse Drug Reactions to the UK ‘Yellow Card Scheme’: Literature Review, Descriptive and Qualitative Analyses, and Questionnaire Surveys,” Health Technology Assessment (Winchester) 15, no. 20 (2011): 1–234. 10.3310/hta15200 21545758

[42] “EVAS – Evaluation and Enhancement of Analyses in Spontaneous Reporting Databases,” https://www.bfarm.de/EN/BfArM/Tasks/Research/Drug‐allergies/_node.html.

[43] D. Dubrall , P. Christ , S. Domgörgen , M. Schmid , and B. Sachs , “Factors Associated With the Completeness of Information Provided in Adverse Drug Reaction Reports of Physicians, Pharmacists and Consumers From Germany,” Scientific Reports 15 (2025): 23751. 40610517 10.1038/s41598-025-07973-9 PMC12229551

