# Supplementary material for: Patterns of Spontaneous Adverse Drug Reaction Reporting in Germany From 2012 to 2021
Source: Pharmacol Res Perspect. 2026 Jun 11;14(3):e70282. doi: 10.1002/prp2.70282 (PMC13260685; doi:10.1002/prp2.70282)
Supplement: Supplementary file 1 — Table S1: Descriptive analysis of the dataset (n = 407 882). Figure S1: Number of reports referring to females, males and unknown sex. Figure S2: Reporting rates of ADR reports referring to females of specific age groups per 10 000 patients with outpatient drug prescriptions per year. Figure S3: Reporting rates of ADR reports referring to males of specific age groups per 10 000 patients with outpatient drug prescriptions per year. Figure S4: Reporting rates of ADR reports referring to patients aged 0–2 years per 10 000 patients with outpatient drug prescriptions per year. Figure S5: Reporting rates of ADR reports referring to patients aged 3–11 years per 10 000 patients with outpatient drug prescriptions per year. Figure S6: Reporting rates of ADR reports referring to patients aged 12–17 years per 10 000 patients with outpatient drug prescriptions per year. Figure S7: Reporting rates of ADR reports referring to patients aged 18–64 years per 10 000 patients with outpatient drug prescriptions per year. Figure S8: Reporting rates of ADR reports referring to patients aged 65–85 years per 10 000 patients with outpatient drug prescriptions per year. Figure S9: Reporting rates of ADR reports referring to patients aged ≥ 86 years per 10 000 patients with outpatient drug prescriptions per year. Figure S10: Number of ADR reports referring to patients of specific age groups per year. Figure S11: Number of ADR reports referring to females of specific age groups per year. Figure S12: Number of ADR reports referring to males of specific age groups per year. Figure S13: Reporting rates of serious ADR reports in total and referring to females and males per 10 000 patients with outpatient drug prescriptions per year. Figure S14: Number of serious ADR reports referring to females, males and unknown sex per year. Figure S15: Reporting rates of serious ADR reports for specific age groups per 10 000 patients with outpatient drug prescriptions per year. Figure S16: Reporting rates of ADR [file PRP2-14-e70282-s001.docx]

**Table S1) Descriptive analysis of the dataset (n= 407,882).**

| Receive year | Number of reports | Females | Males | Unknown | Mean Age  (± SD) | Reports with unknown age of the patient | Reports classified as serious | Reports classified as non-serious | Reports from physicians | Reports from consumers |
| --- | --- | --- | --- | --- | --- | --- | --- | --- | --- | --- |
| 2012 | 21831 | 11858 (54.3%) | 8569 (39.3%) | 1404 (6.4%) | 54.6 (± 21.2) | 6434 (29.5%) | 17048 (78.1%) | 4783 (21.9%) | 11776 (53.9%) | 3381 (15.5%) |
| 2013 | 22198 | 12411 (55.9%) | 8672 (39.1%) | 1115 (5.0%) | 54.4 (± 21.7) | 6215 (28.0%) | 18467 (83.2%) | 3731 (16.8%) | 11694 (52.7%) | 4208 (19.0%) |
| 2014 | 21408 | 11955 (55.8%) | 8501 (39.7%) | 952 (4.4%) | 54.4 (± 22.1) | 6021 (28.1%) | 17299 (80.1%) | 4109 (19.2%) | 10872 (50.8%) | 3758 (17.6%) |
| 2015 | 20940 | 11660 (55.7%) | 8374 (40.0%) | 906 (4.3%) | 54.7 (± 21.7) | 6110 (29.2%) | 16059 (76.7%) | 4881 (23.3%) | 10658 (50.9%) | 4092 (19.5%) |
| 2016 | 24075 | 13507 (56.1%) | 9661 (40.1%) | 907 (3.8%) | 54.1 (± 21.8) | 7189 (29.9%) | 17730 (73.6%) | 6345 (26.4%) | 12045 (50.0%) | 5329 (22.1%) |
| 2017 | 31520 | 17768 (56.4%) | 12289 (39.0%) | 1463 (4.6%) | 54.3 (± 21.4) | 10807 (34.3%) | 18077 (57.4%) | 13443 (42.6%) | 13149 (41.7%) | 8622 (27.4%) |
| 2018 | 69955 | 41440 (59.2%) | 24877 (35.6%) | 3638 (5.2%) | 54.8 (± 21.4) | 28486 (40.7%) | 17884 (25.6%) | 52071 (74.4%) | 21228 (30.3%) | 25658 (36.7%) |
| 2019 | 72444 | 44596 (61.6%) | 24820 (34.3%) | 3028 (4.2%) | 53.1 (± 21.5) | 30124 (41.6%) | 18639 (25.7%) | 53805 (74.3%) | 20862 (28.8%) | 30363 (41.9%) |
| 2020 | 69079 | 42853 (62.0%) | 24083 (34.9%) | 2143 (3.1%) | 52.1 (± 21.7) | 28251 (40.9%) | 16825 (24.4%) | 52254 (75.6%) | 16931 (24.5%) | 35801 (51.8%) |
| 2021 | 54432 | 32959 (60.6%) | 19529 (35.9%) | 1944 (3.6%) | 53.0 (± 21.9) | 21949 (40.3%) | 13618 (25.0%) | 40814 (75.0%) | 13192 (24.2%) | 27271 (50.1%) |

Table S1 shows the absolute number of spontaneous ADR reports per year in total, stratified by sex, seriousness and reporter types.

Figure S1) Number of reports referring to females, males and unknown sex.


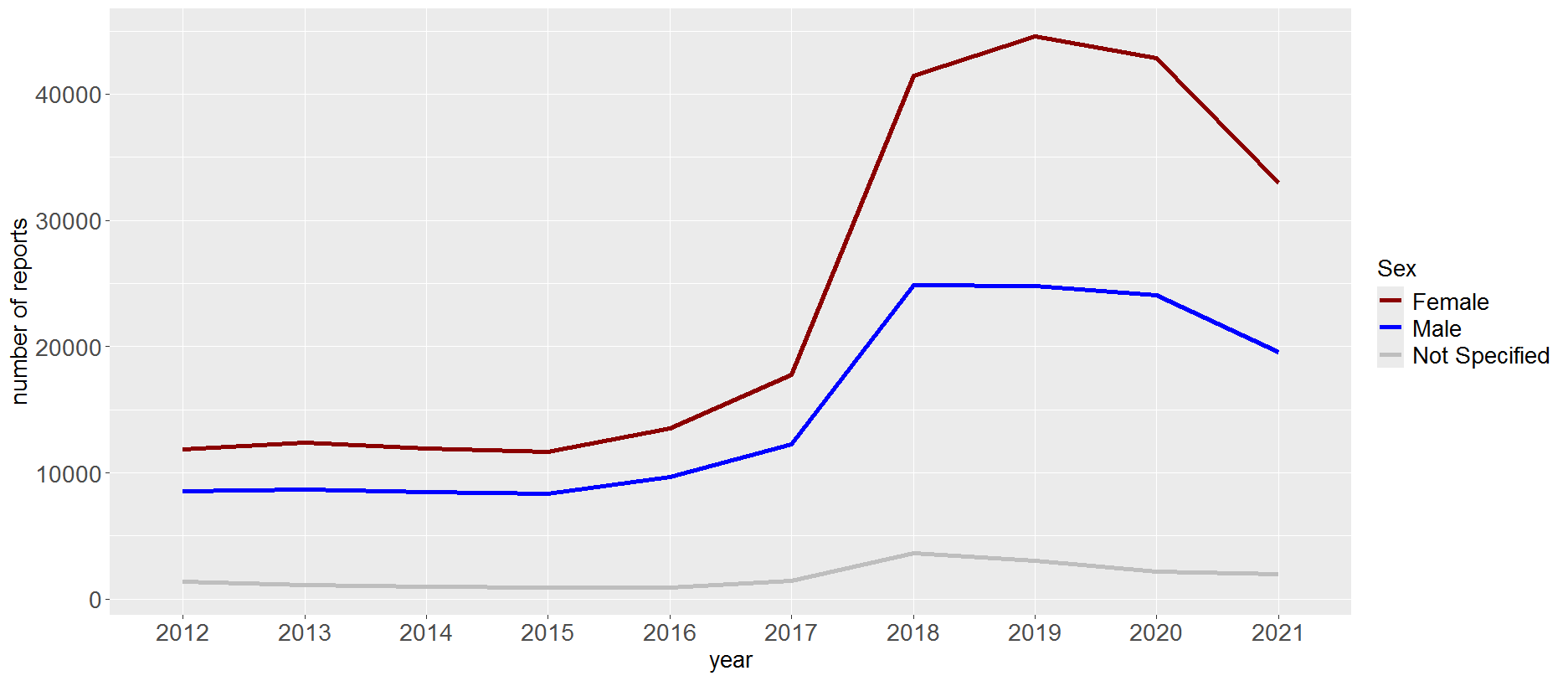


Figure S1 shows the number of ADR reports referring to females, males and sex not specified without considering the number of patients with drug prescriptions.

Figure S2) Reporting rates of ADR reports referring to females of specific age groups per 10,000 patients with outpatient drug prescriptions per year.


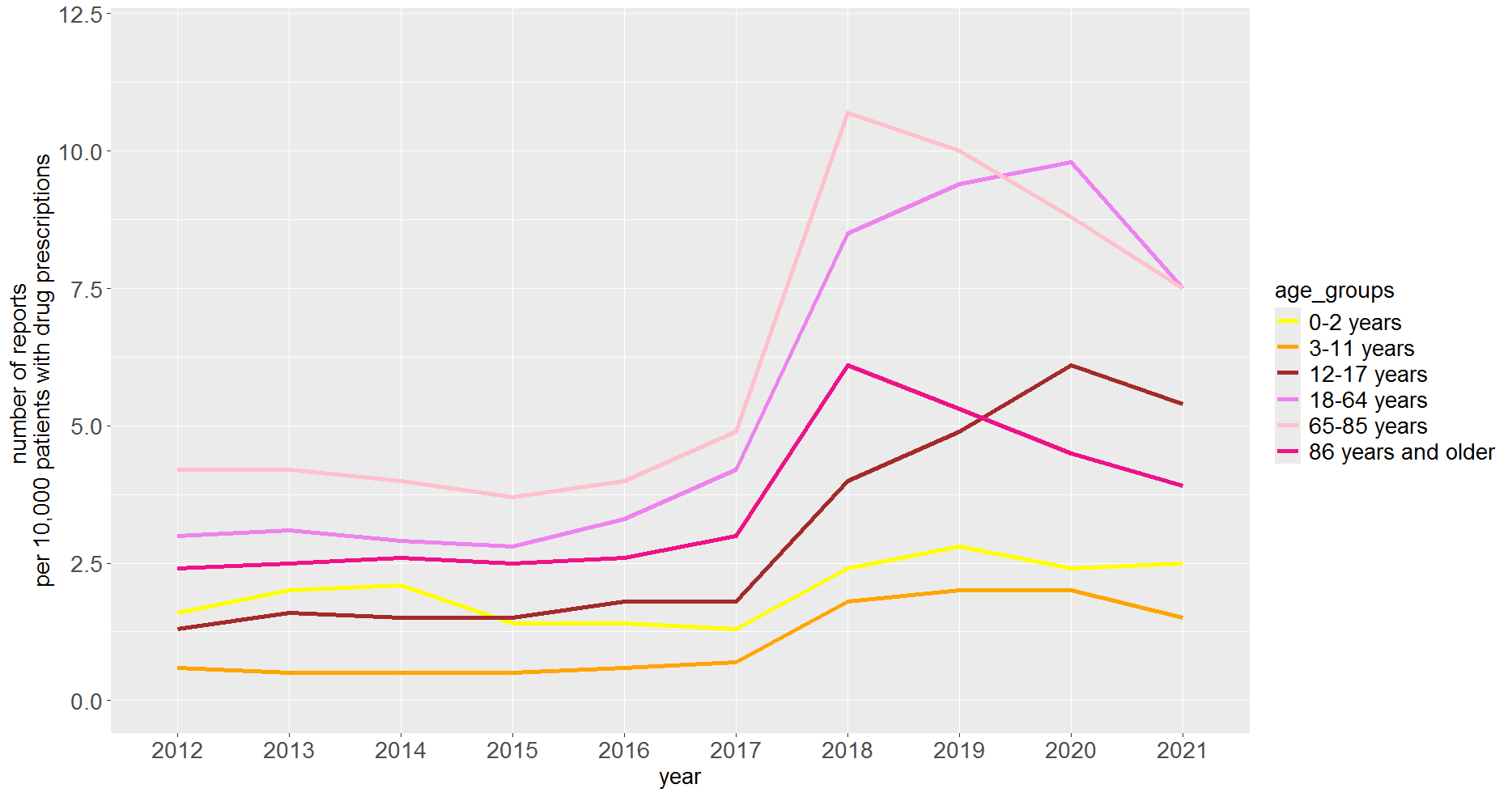


Figure S2 shows the reporting rates of ADR reports referring to females of specific age groups per year.

Figure S3) Reporting rates of ADR reports referring to males of specific age groups per 10,000 patients with outpatient drug prescriptions per year.


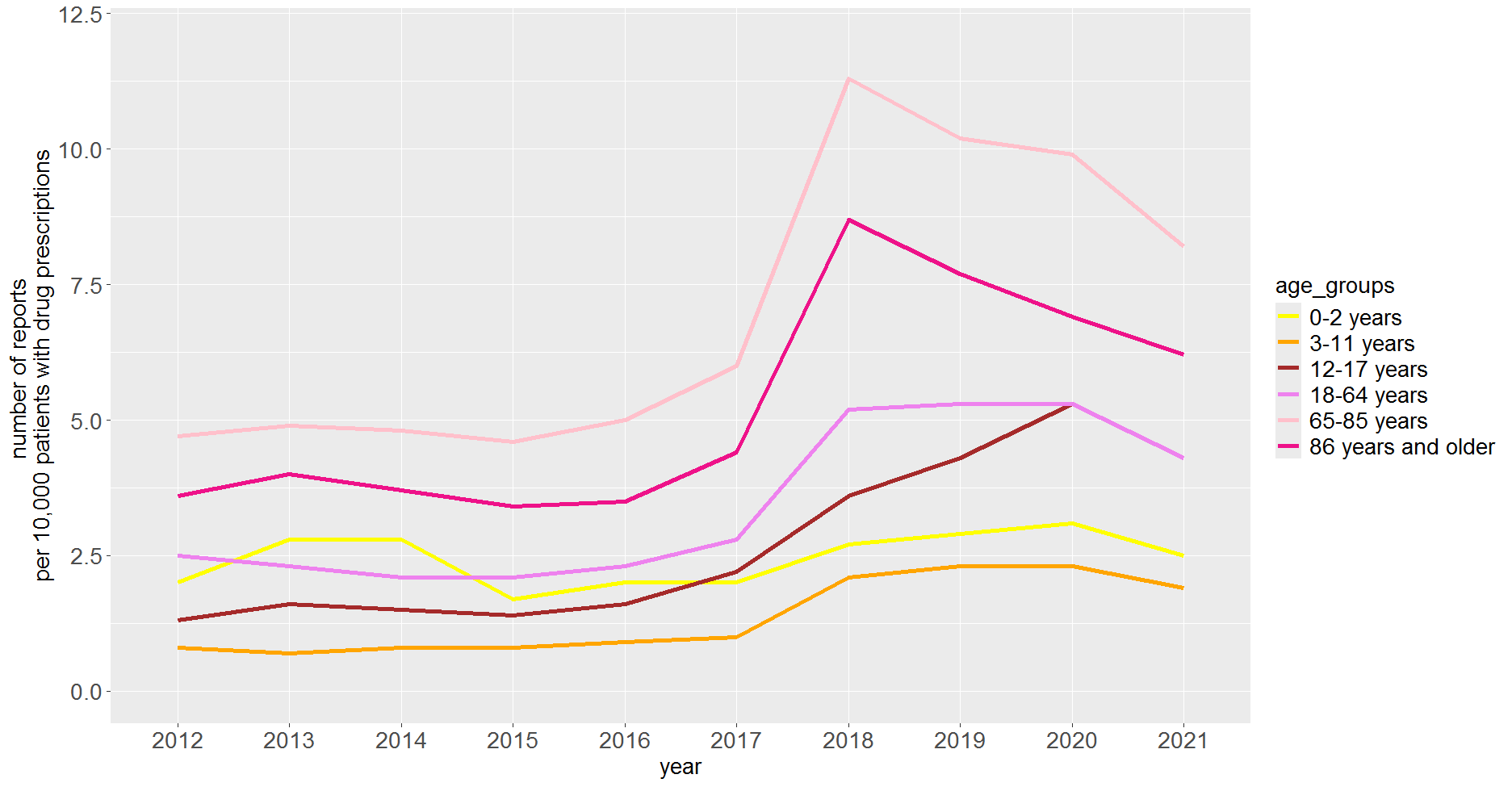


Figure S3 shows the reporting rates of ADR reports referring to males of specific age groups per year.

Figure S4) Reporting rates of ADR reports referring to patients aged 0-2 years per 10,000 patients with outpatient drug prescriptions per year.


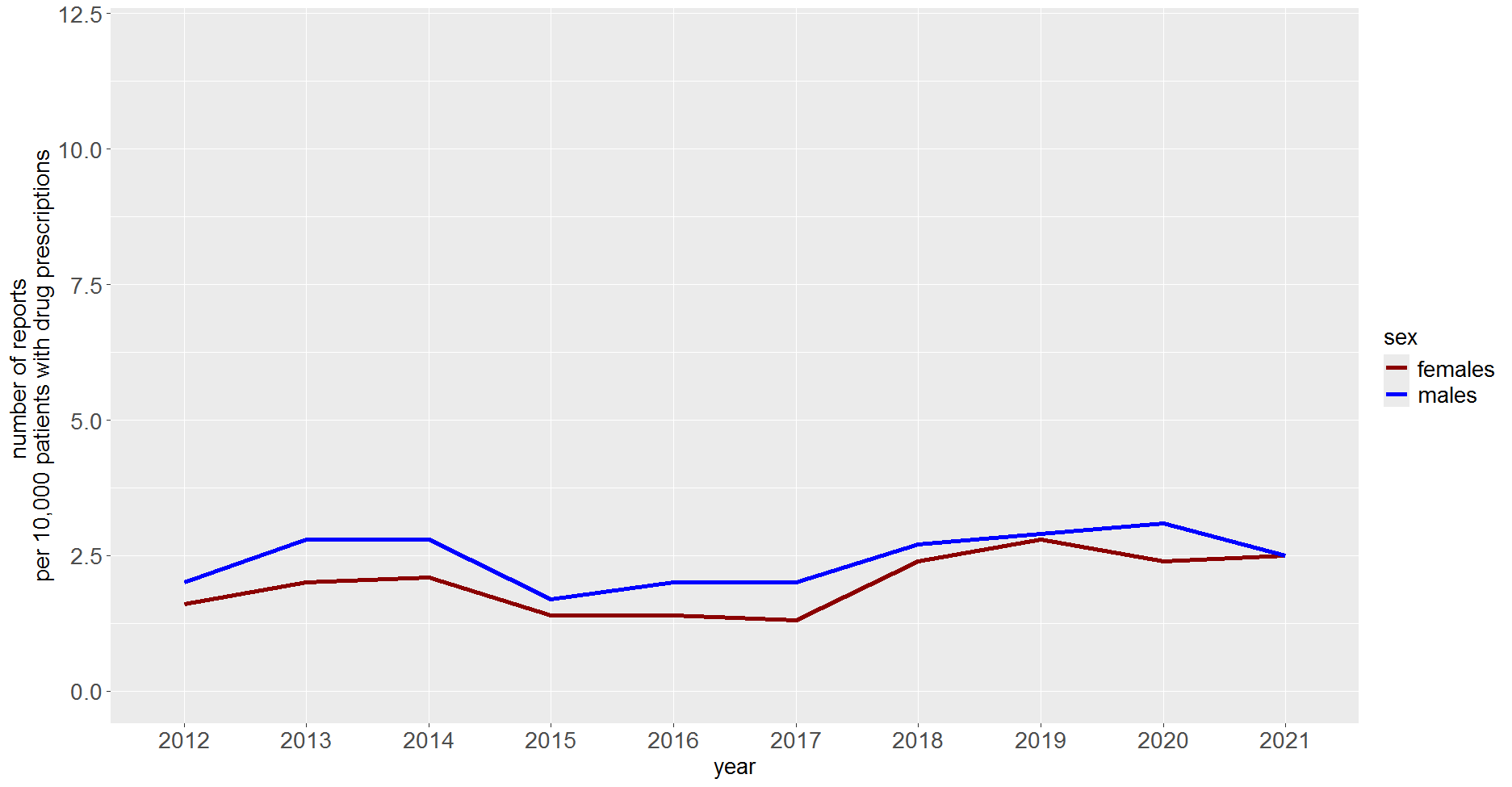


Figure S4 shows the reporting rates of ADR reports referring to patients aged 0-2 years per year.

Figure S5) Reporting rates of ADR reports referring to patients aged 3-11 years per 10,000 patients with outpatient drug prescriptions per year.


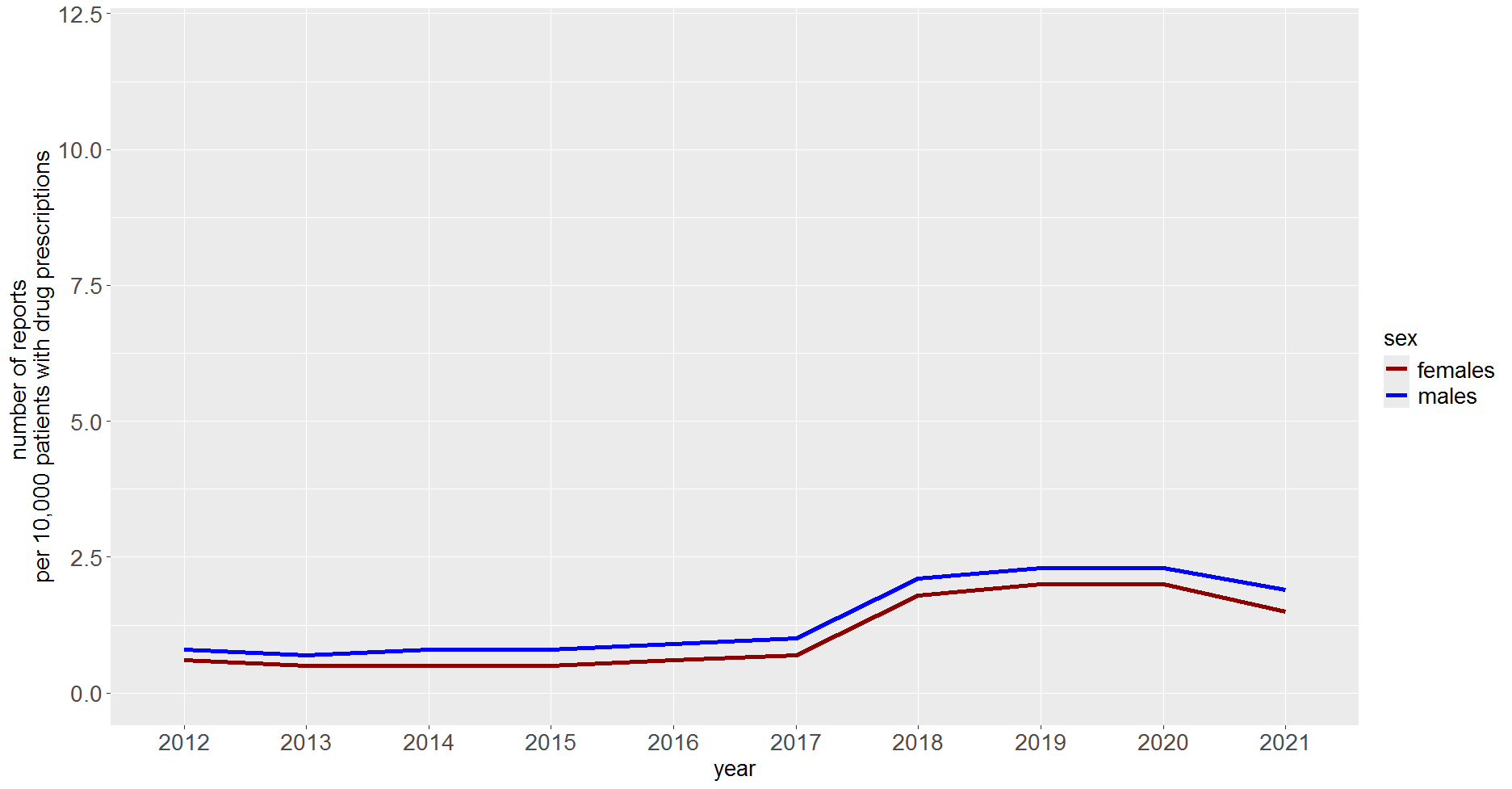


Figure S5 shows the reporting rates of ADR reports referring to patients aged 3-11 years per year.

Figure S6) Reporting rates of ADR reports referring to patients aged 12-17 years per 10,000 patients with outpatient drug prescriptions per year.


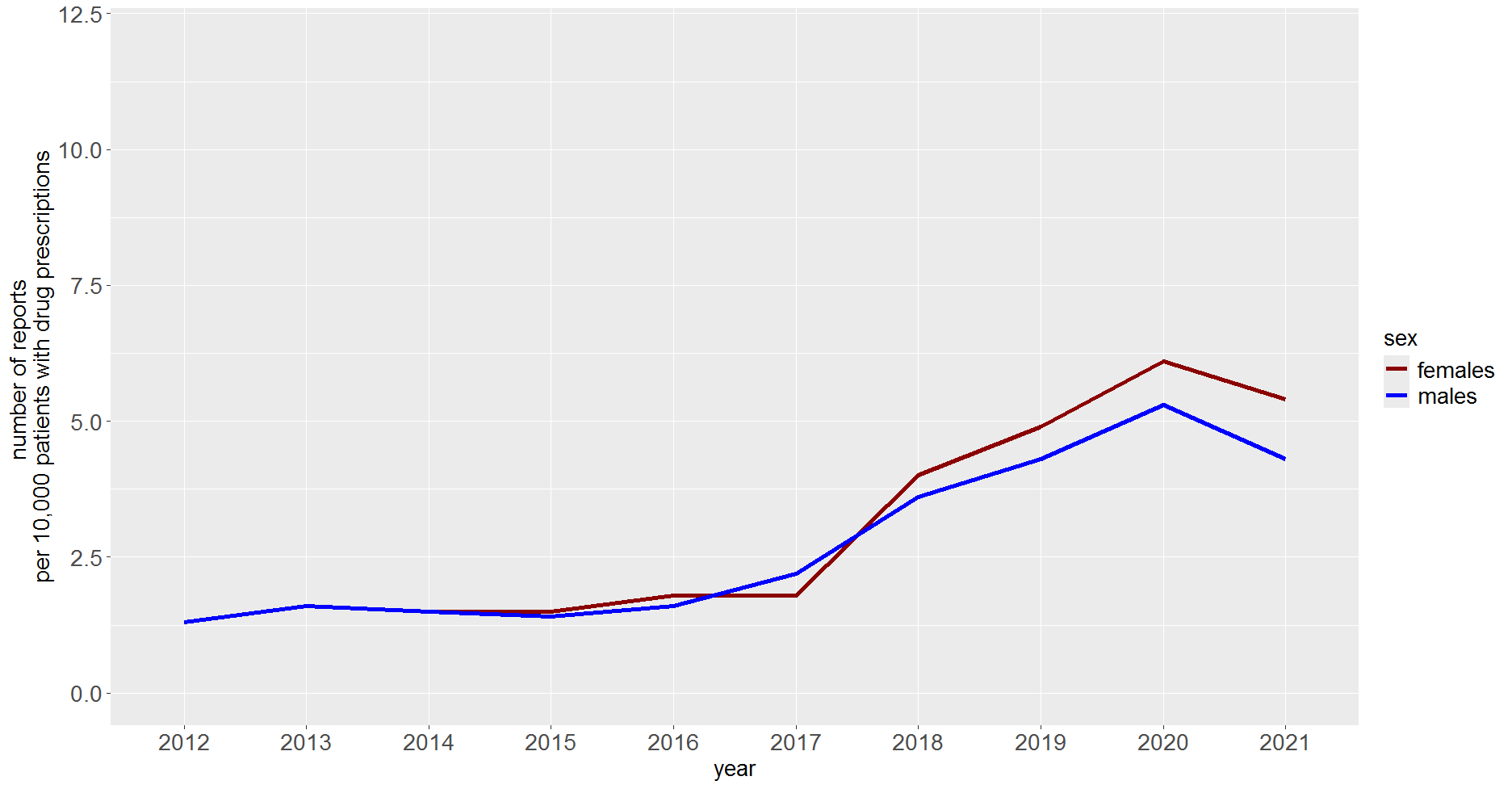


Figure S6 shows the reporting rates of ADR reports referring to patients aged 12-17 years per year.

Figure S7) Reporting rates of ADR reports referring to patients aged 18-64 years per 10,000 patients with outpatient drug prescriptions per year.


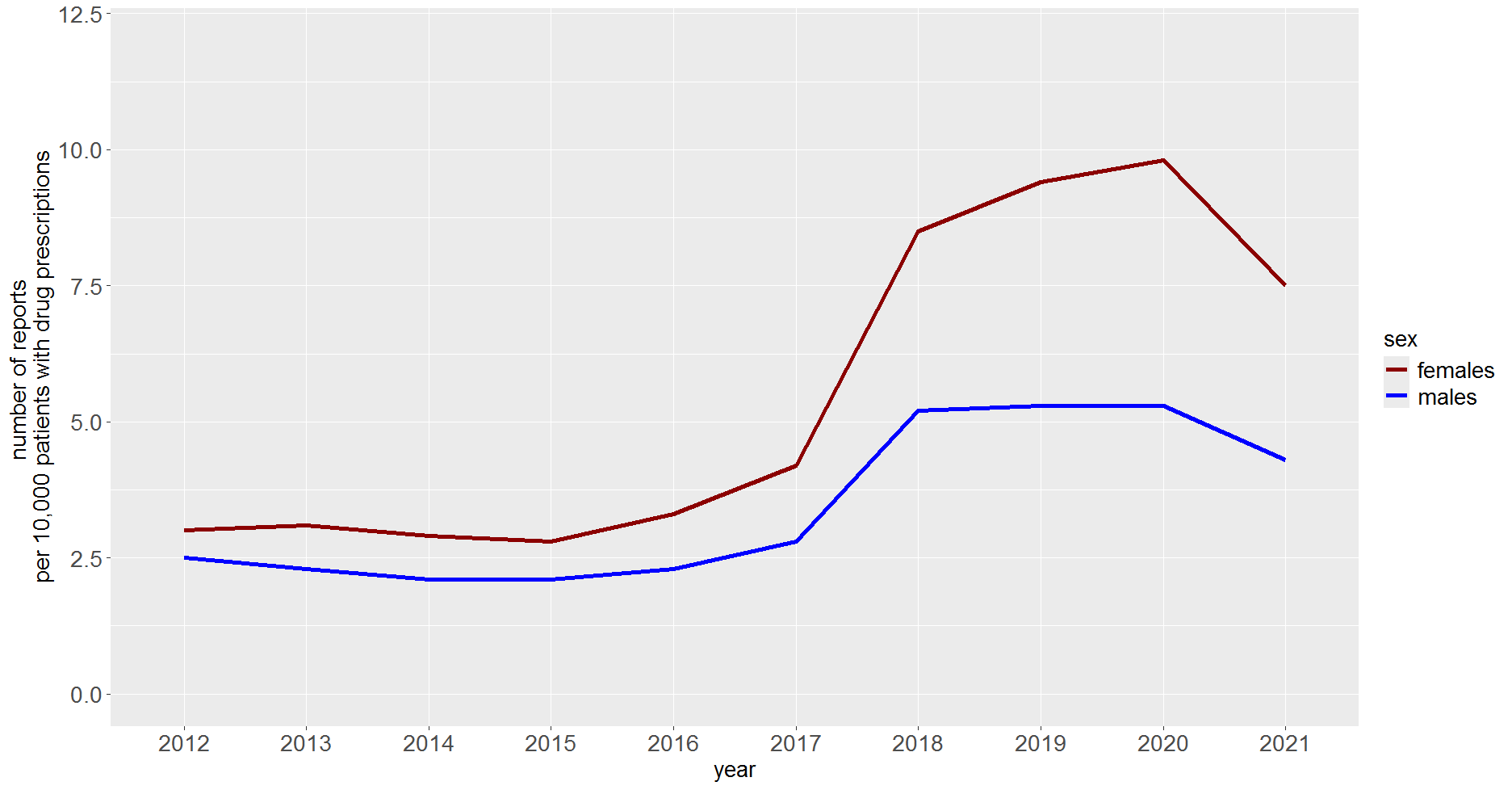


Figure S7 shows the reporting rates of ADR reports referring to patients aged 18-64 years per year.

Figure S8) Reporting rates of ADR reports referring to patients aged 65-85 years per 10,000 patients with outpatient drug prescriptions per year.


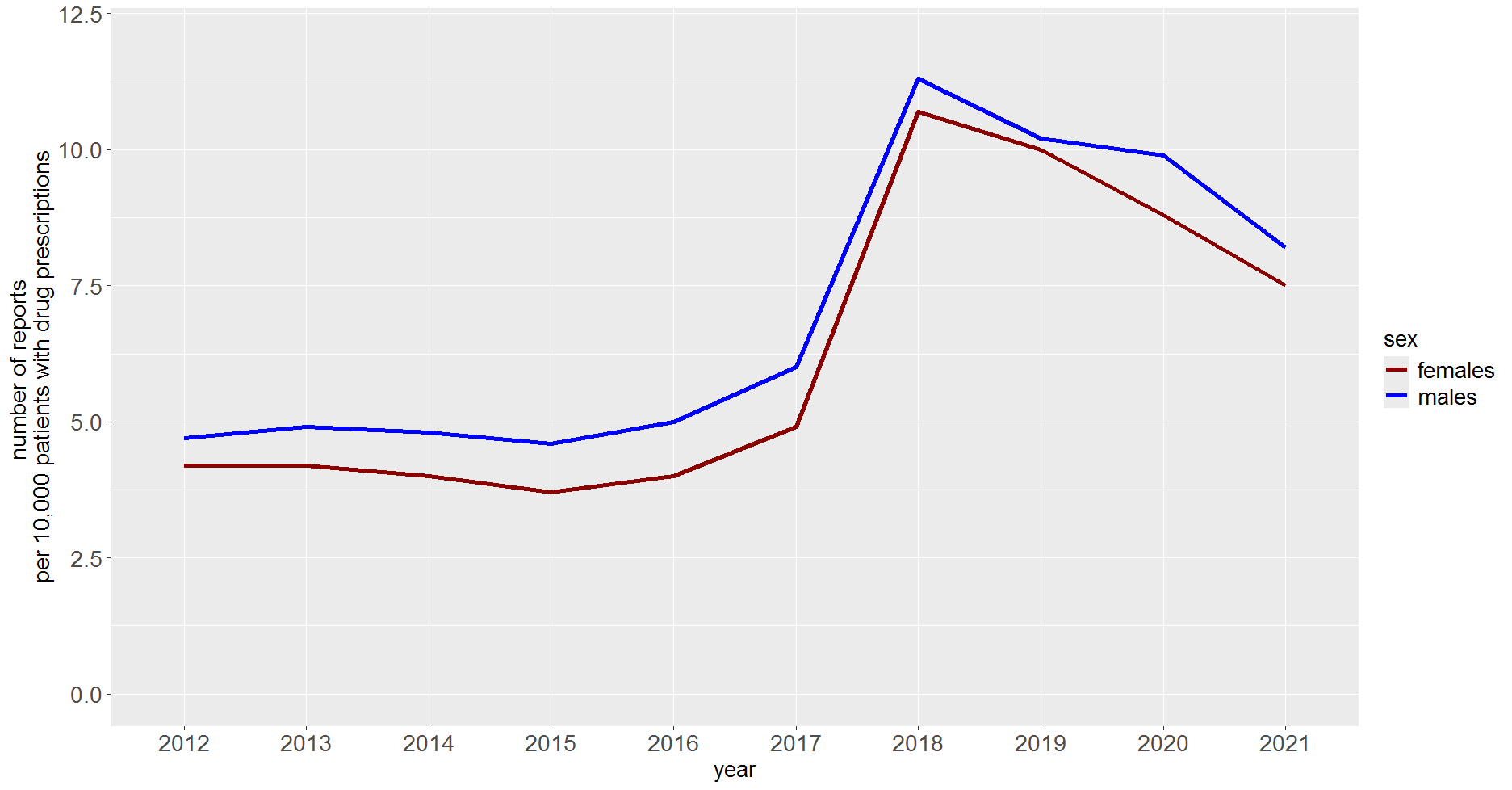


Figure S8 shows the reporting rates of ADR reports referring to patients aged 65-85 years per year.

Figure S9) Reporting rates of ADR reports referring to patients aged ≥ 86 years per 10,000 patients with outpatient drug prescriptions per year.


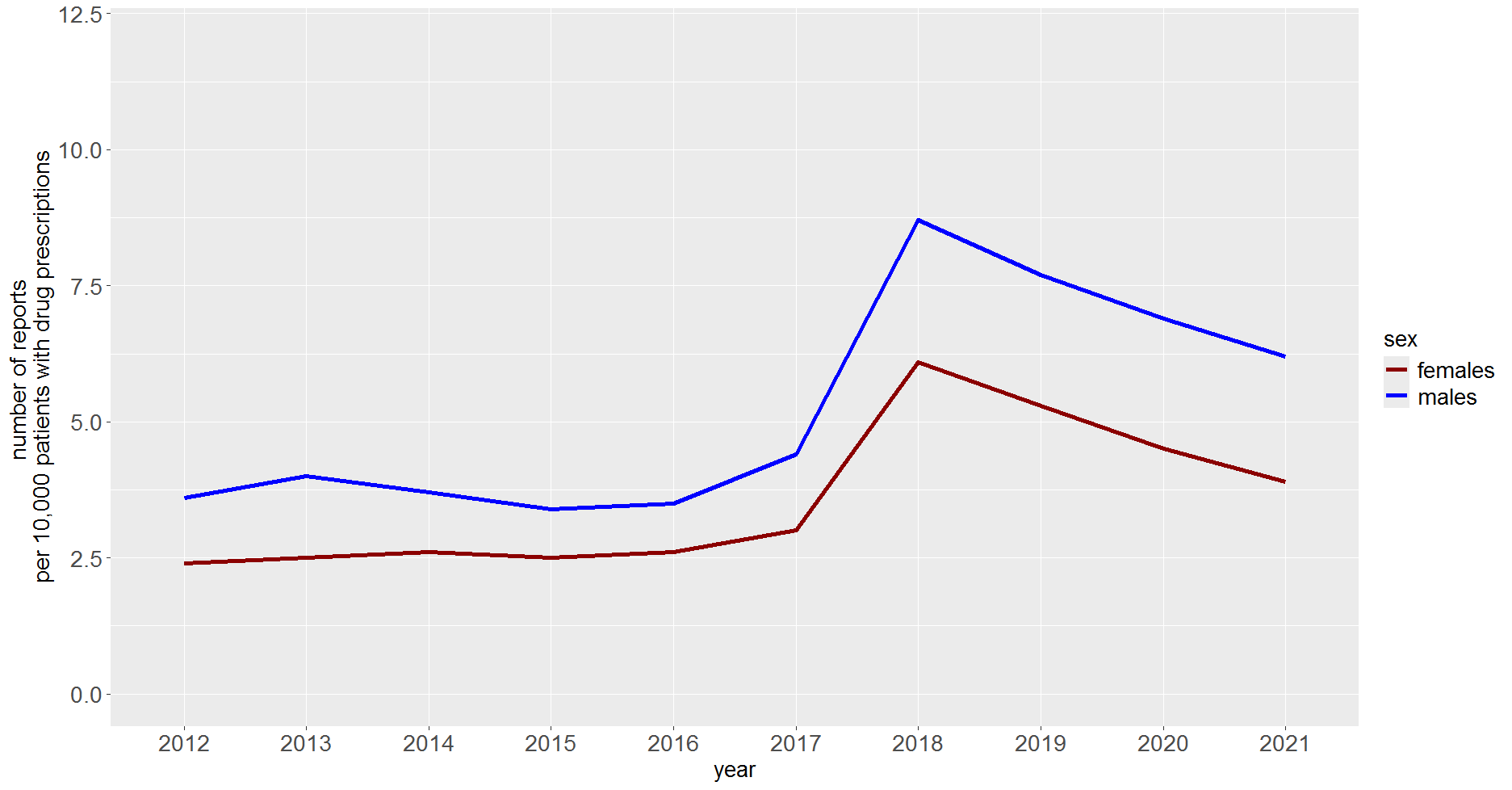


Figure S9 shows the reporting rates of ADR reports referring to patients aged ≥ 86 years per year.

Figure S10) Number of ADR reports referring to patients of specific age groups per year.


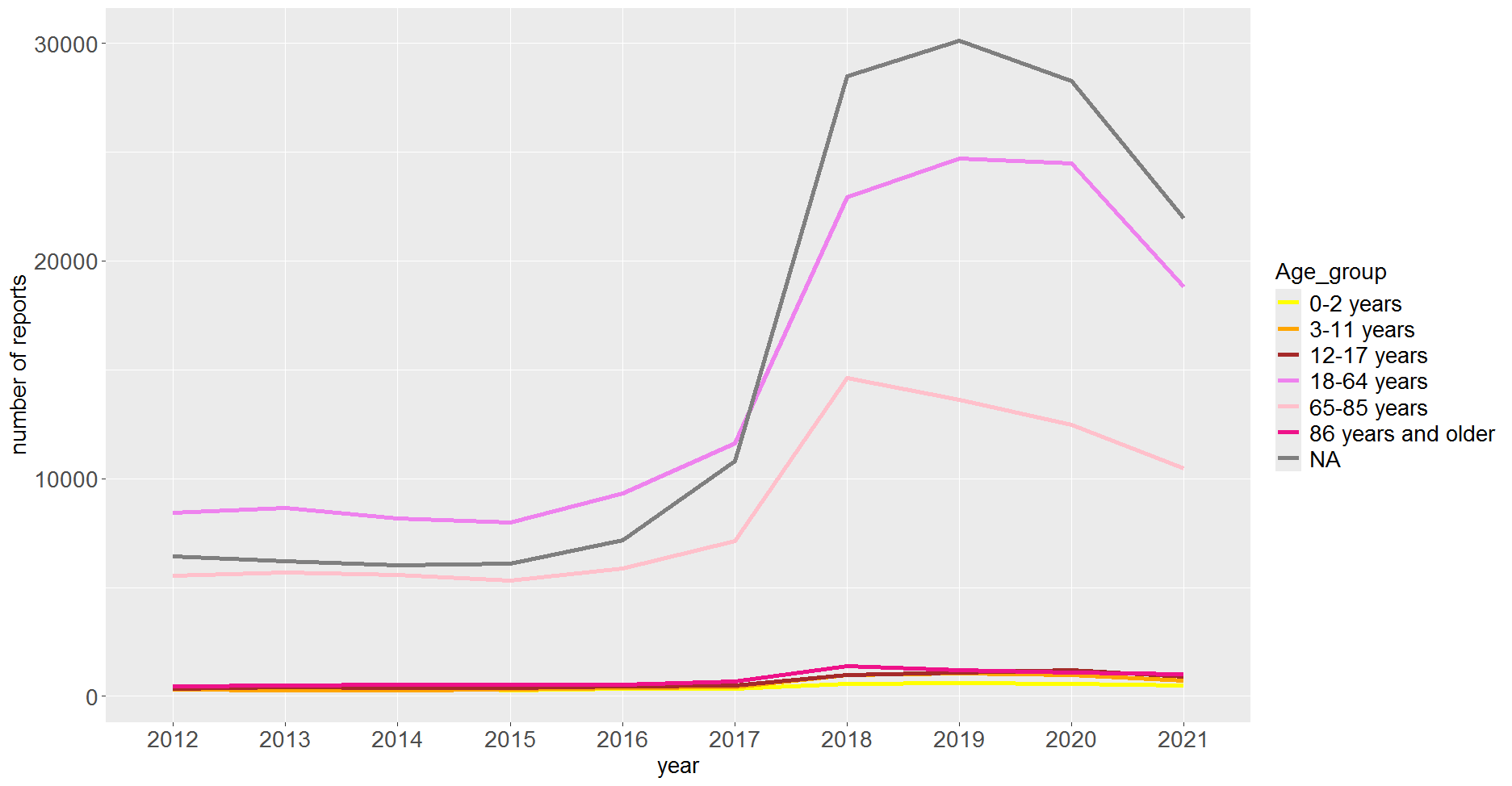


Figure S10 shows the raw number of ADR reports referring to specific age groups per year.

Figure S11) Number of ADR reports referring to females of specific age groups per year.


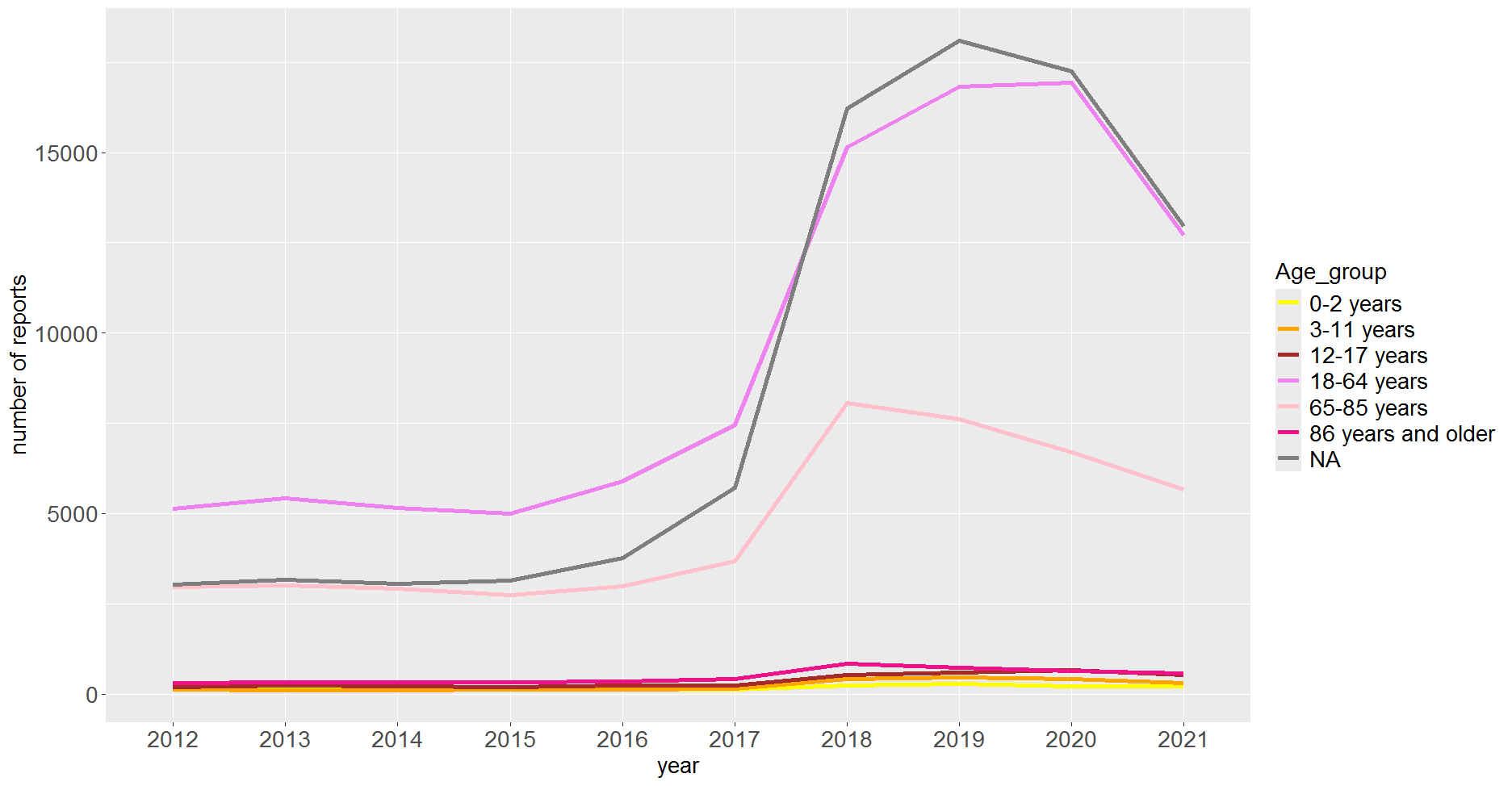


Figure S11 shows the raw number of ADR reports referring to females of specific age groups per year.

Figure S12) Number of ADR reports referring to males of specific age groups per year.


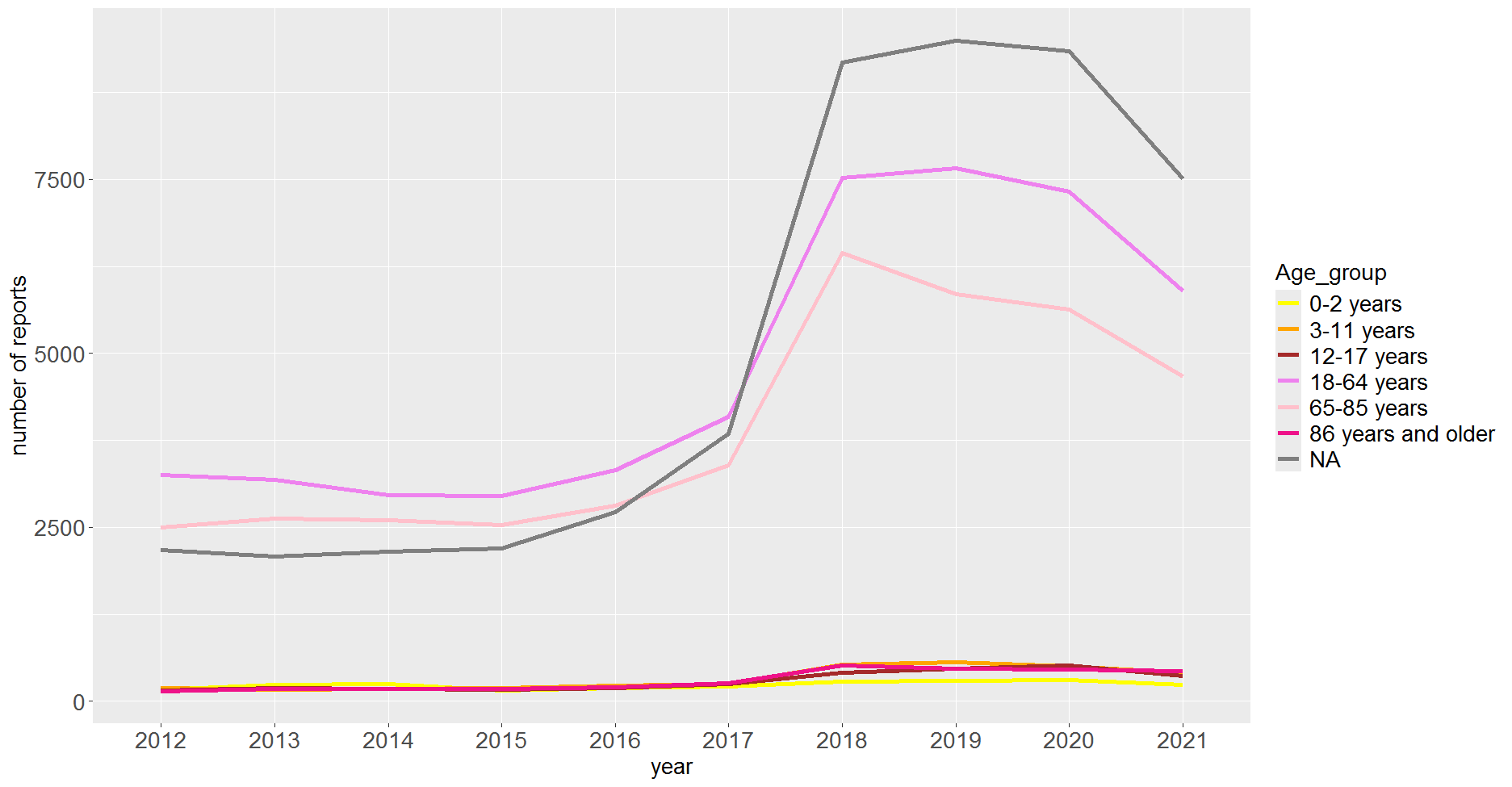


Figure S12 shows the raw number of ADR reports referring to males of specific age groups per year.

Figure S13) Reporting rates of serious ADR reports in total and referring to females and males per 10,000 patients with outpatient drug prescriptions per year.


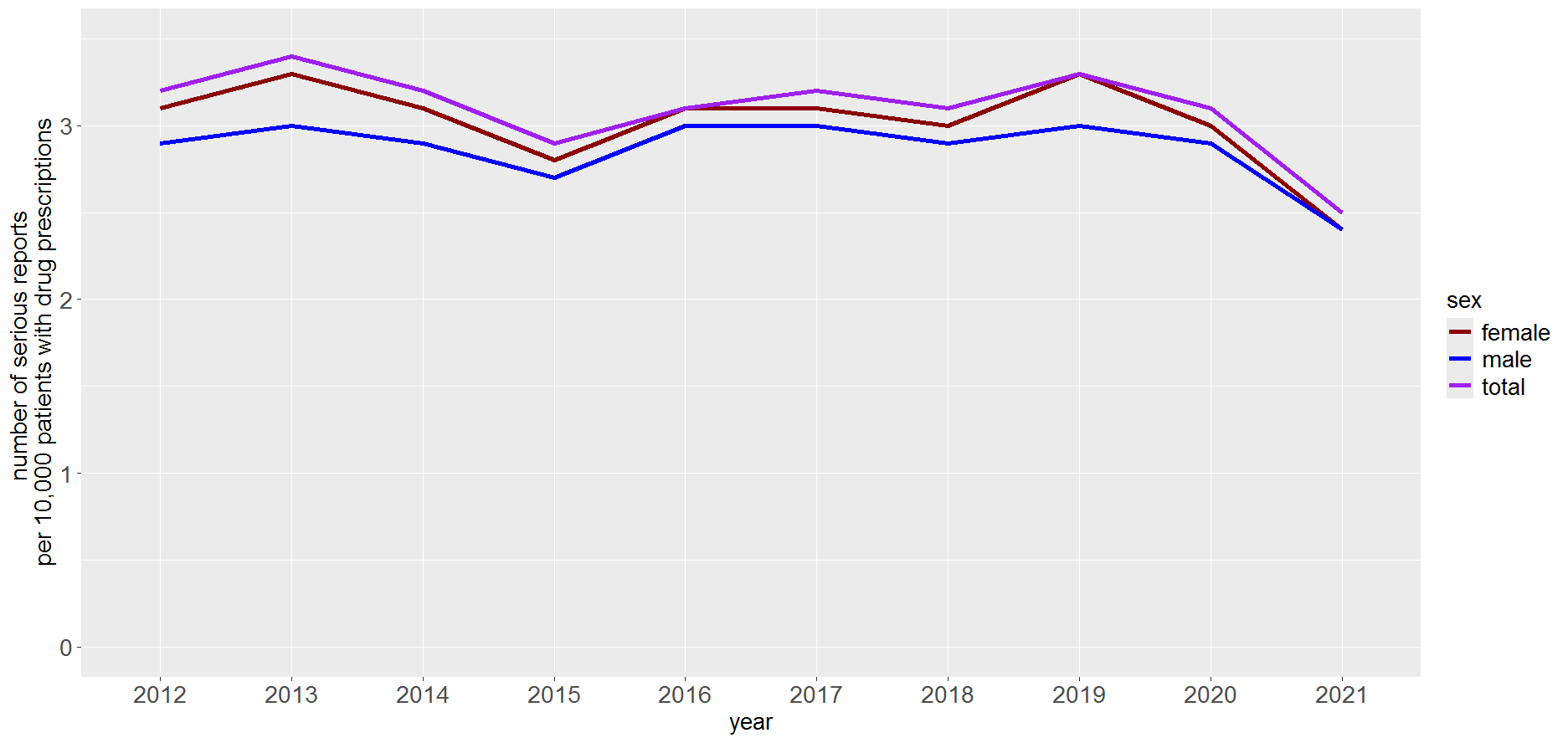


Figure S13 shows the reporting rates of serious ADR reports in total and referring to females and males. Note that, ADR reports with unknown sex are also included in the reporting rates of total ADR reports.

Figure S14) Number of serious ADR reports referring to females, males and unknown sex per year.


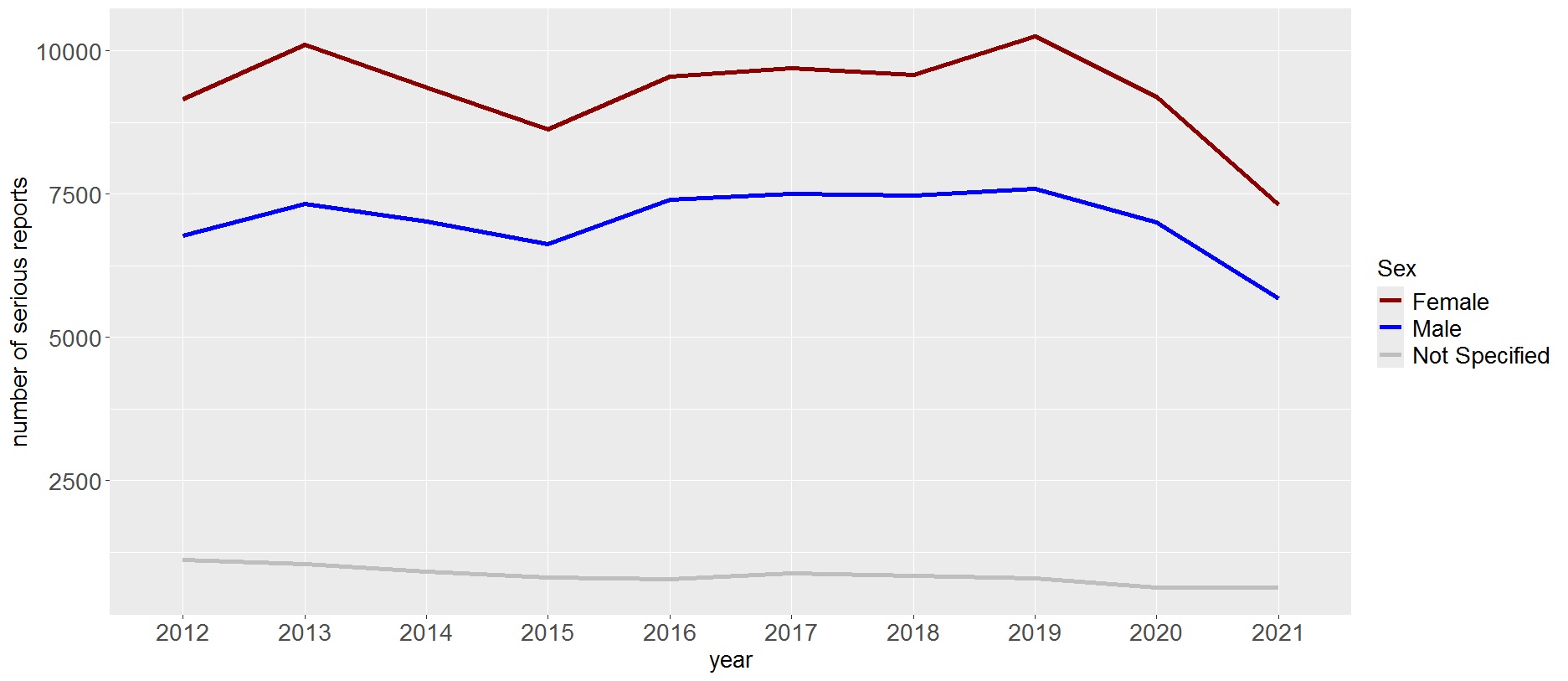


Figure S14 shows the number of serious ADR reports referring to females, males and unknown sex per year.

Figure S15) Reporting rates of serious ADR reports for specific age groups per 10,000 patients with outpatient drug prescriptions per year.


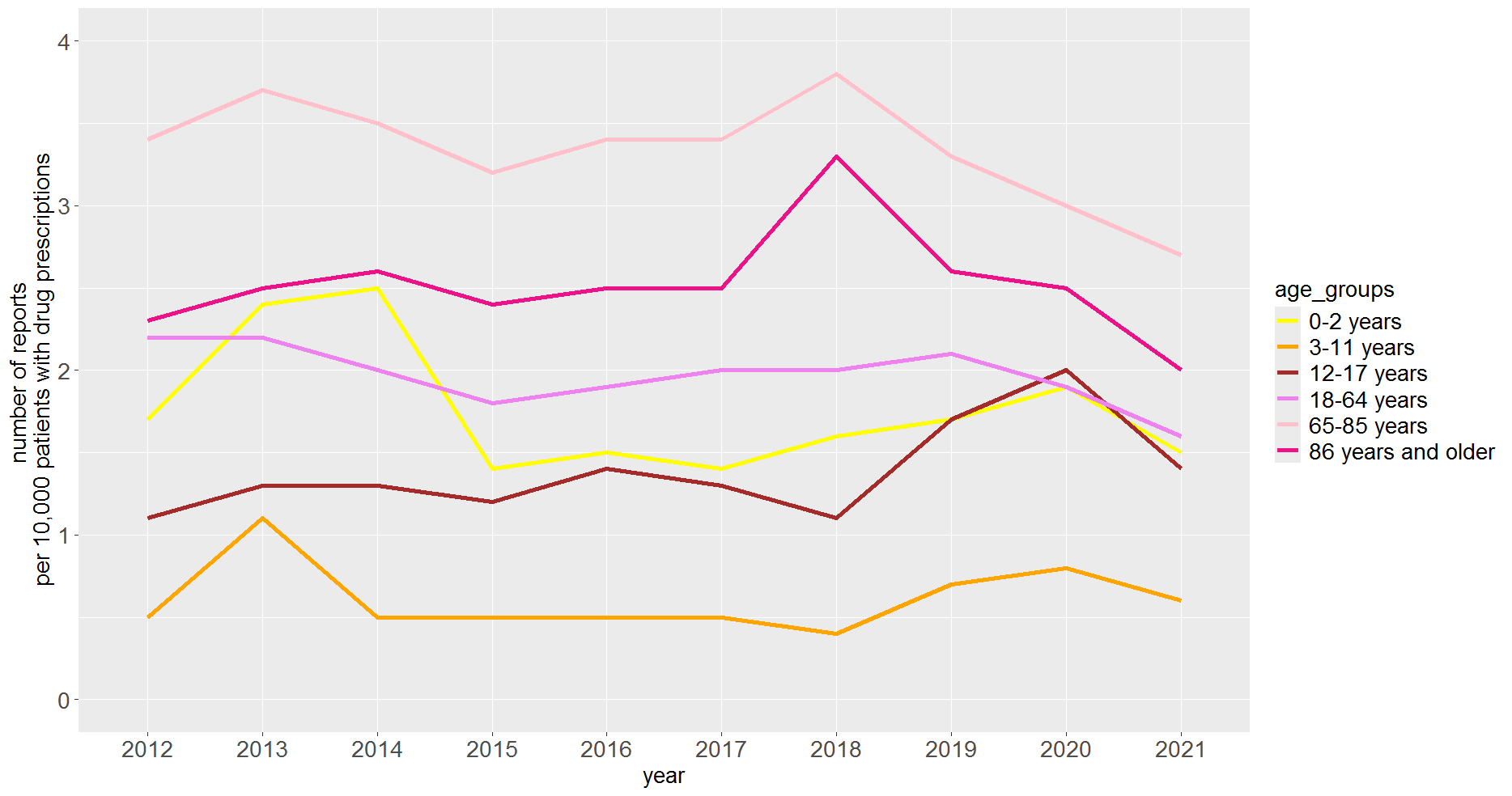


Figure S15 shows the reporting rates of serious ADR reports for specific age groups per year.

Figure S16) Reporting rates of ADR reports coded with hospitalization or prolongation thereof for specific age groups per 10,000 patients with outpatient drug prescriptions per year.


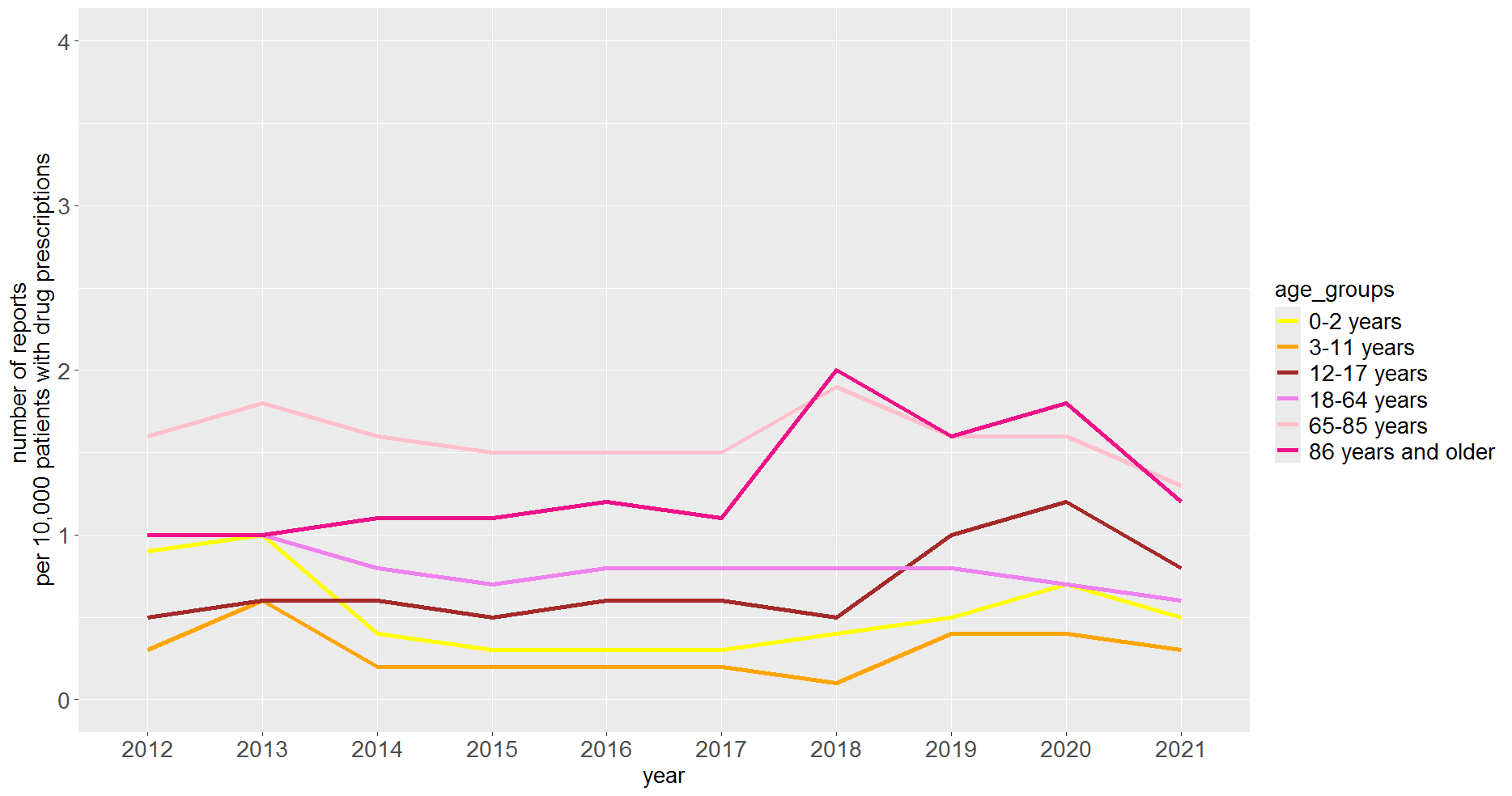


Figure S16 shows the reporting rates of ADR reports coded with hospitalization or prolongation thereof for specific age groups per year.

Figure S17) Reporting rates of non-serious ADR reports in total and referring to females and males per 10,000 patients with outpatient drug prescriptions per year.


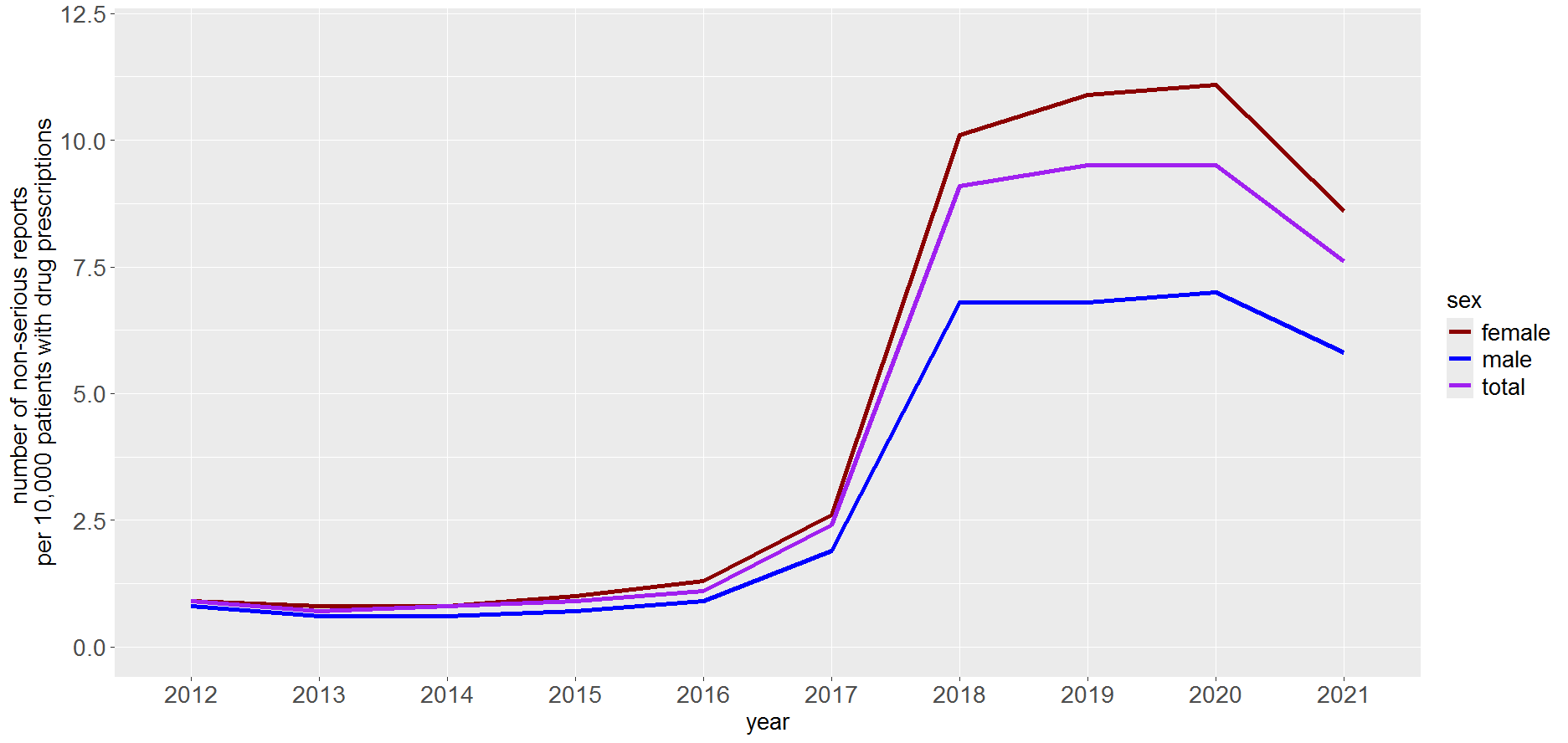


Figure S17 shows the reporting rates of non-serious ADR reports in total and referring to females and males.

Figure S18) Number of non-serious ADR reports referring to females, males and unknown sex per 10,000 patients with outpatient drug prescriptions per year.


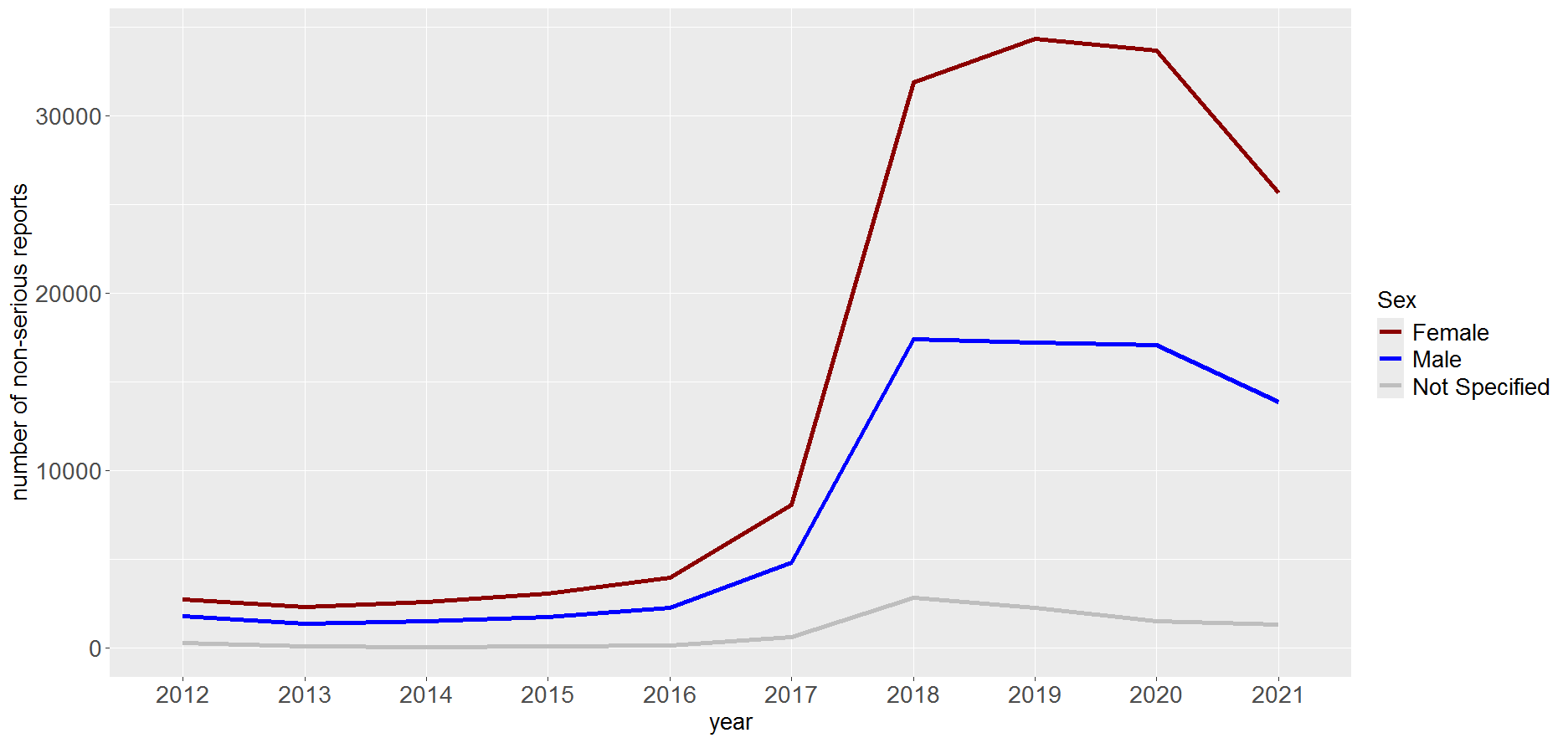


Figure S18 shows the number of non-serious ADR reports referring to females, males and unknown sex per year.

Figure S19) Reporting rates of non-serious ADR reports from physicians and consumers referring to females and males per 10,000 patients with outpatient drug prescriptions per year.


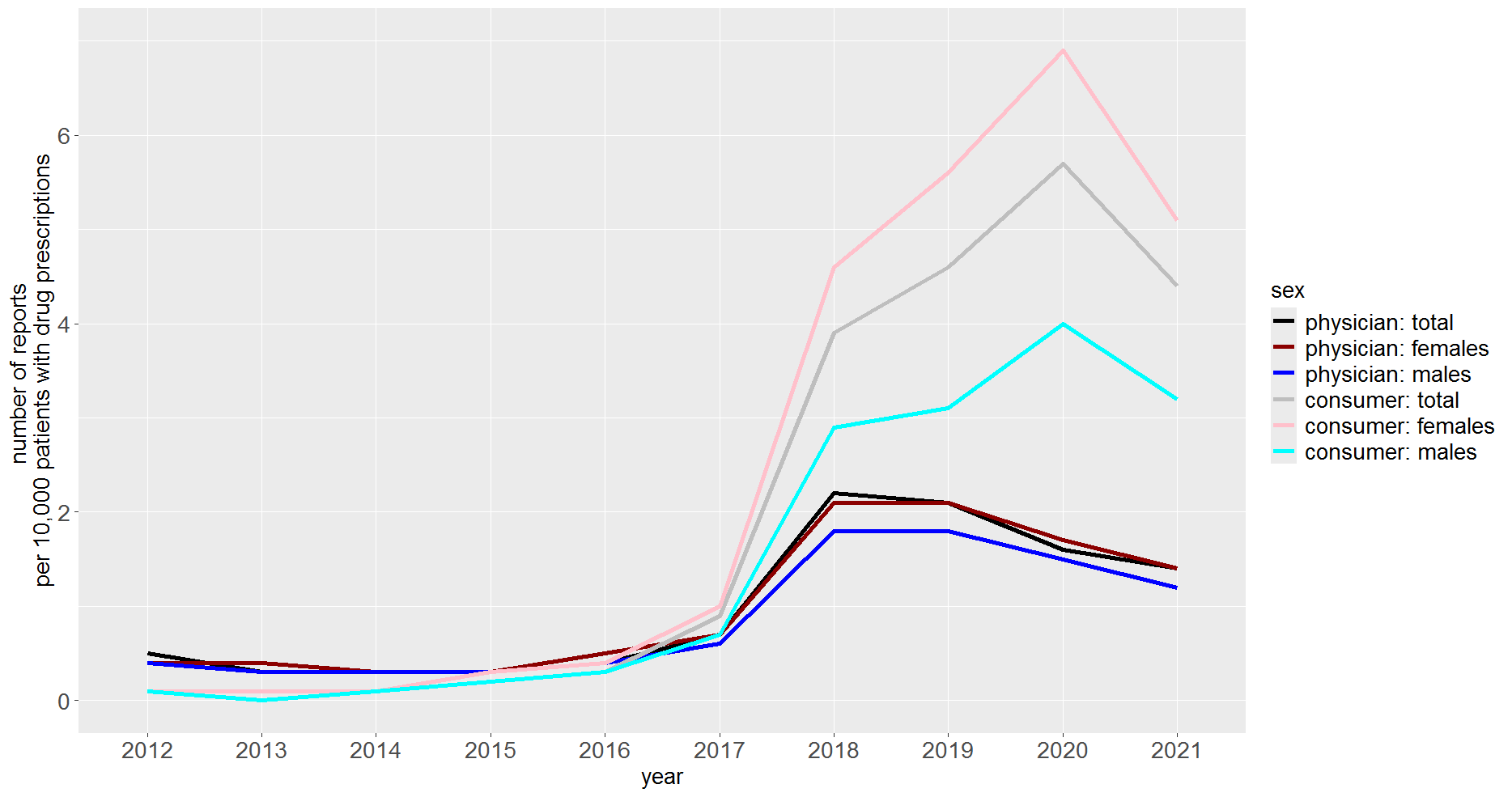


Figure S19 shows the reporting rates of non-serious ADR reports from physicians and consumers in total and referring to females and males.

Figure S20) Reporting rates of serious ADR reports from physicians and consumers referring to females and males per 10,000 patients with outpatient drug prescriptions per year.


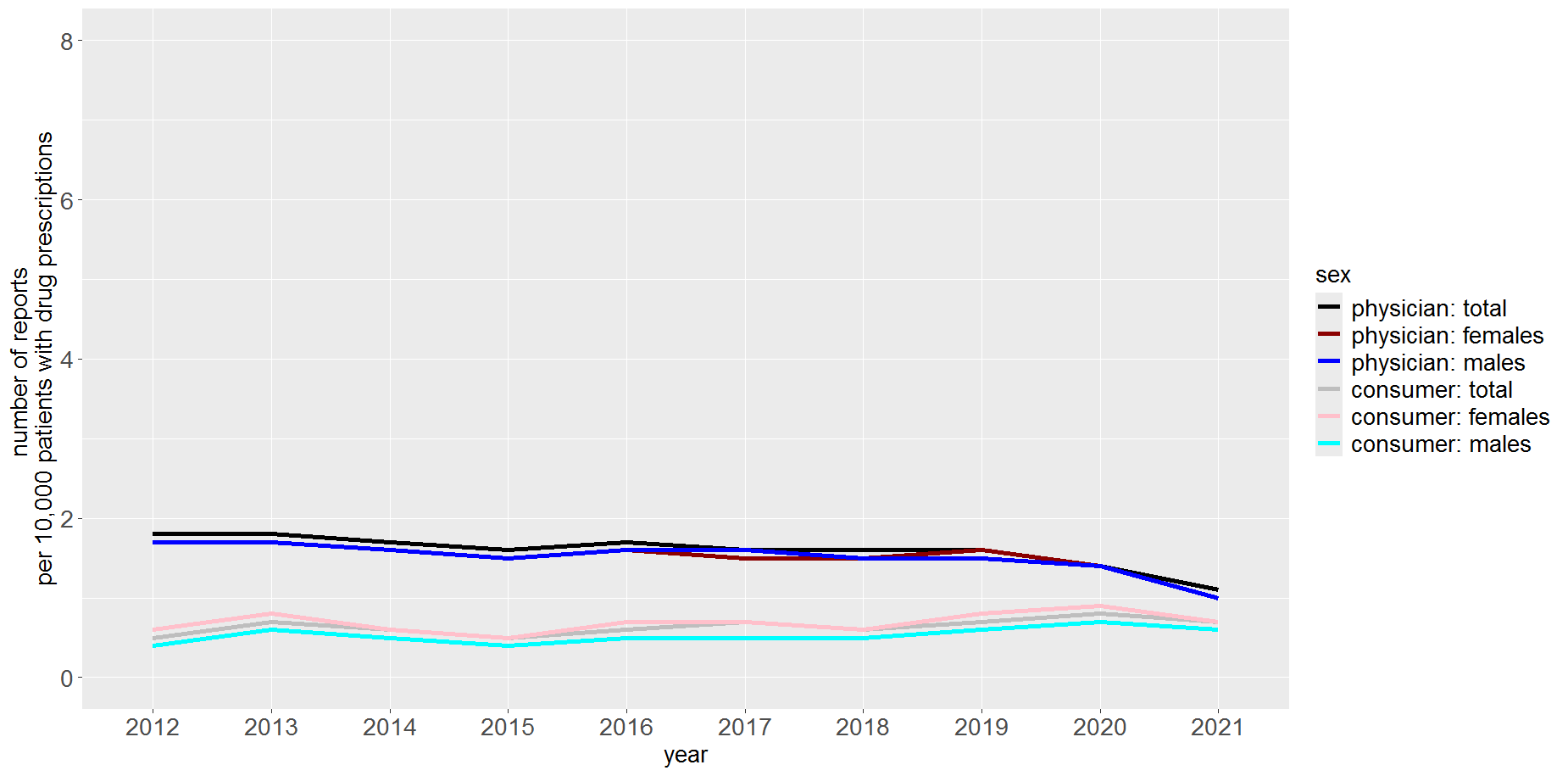


Figure S20 shows the reporting rates of serious ADR reports from physicians and consumers in total and referring to females and males.

Figure S21) Number of ADR reports from consumers


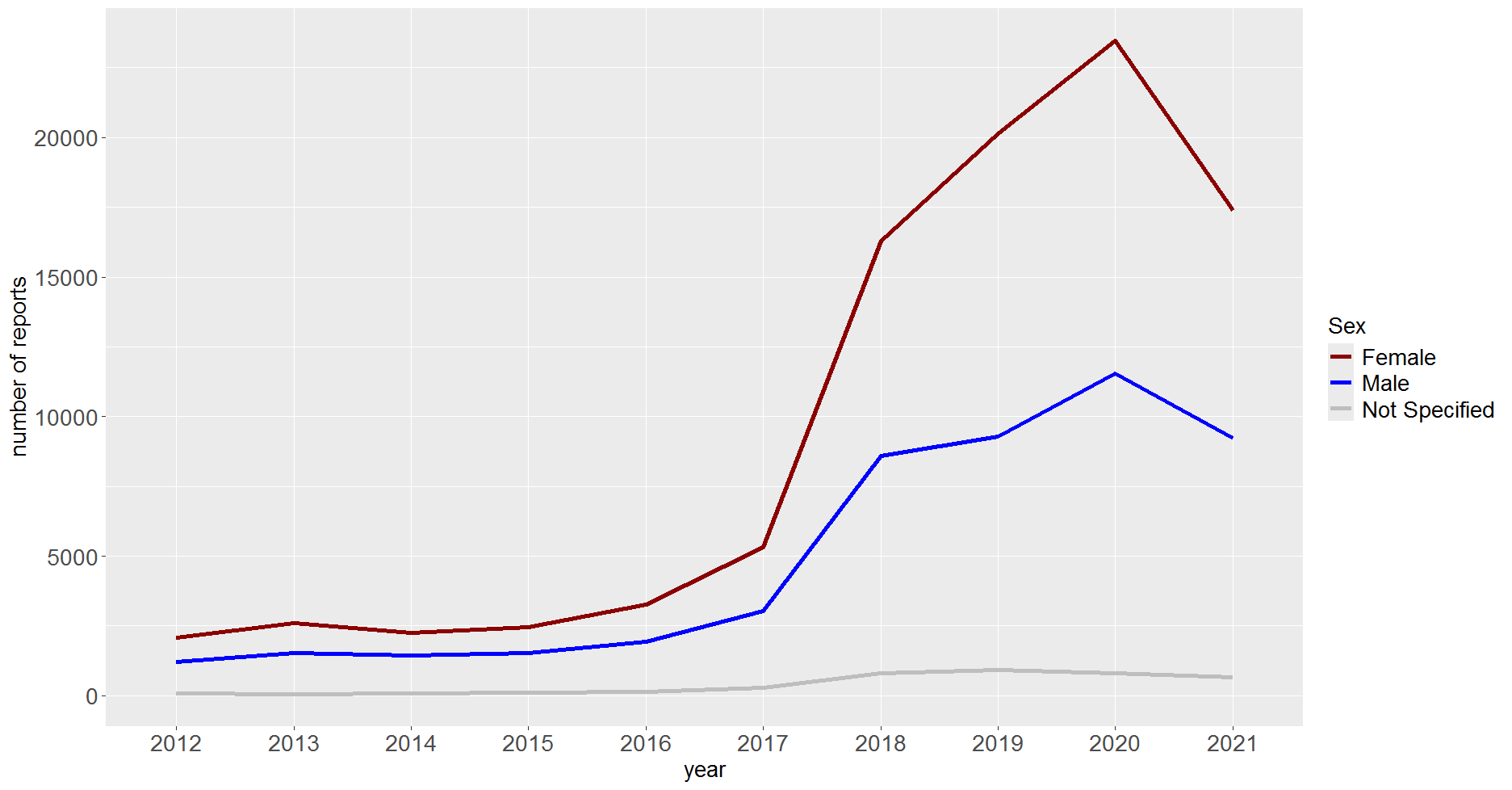


Figure S21 shows the raw number of ADR reports from consumers referring to females, males and unknown sex per year.

Figure S22) Number of ADR reports from physicians.


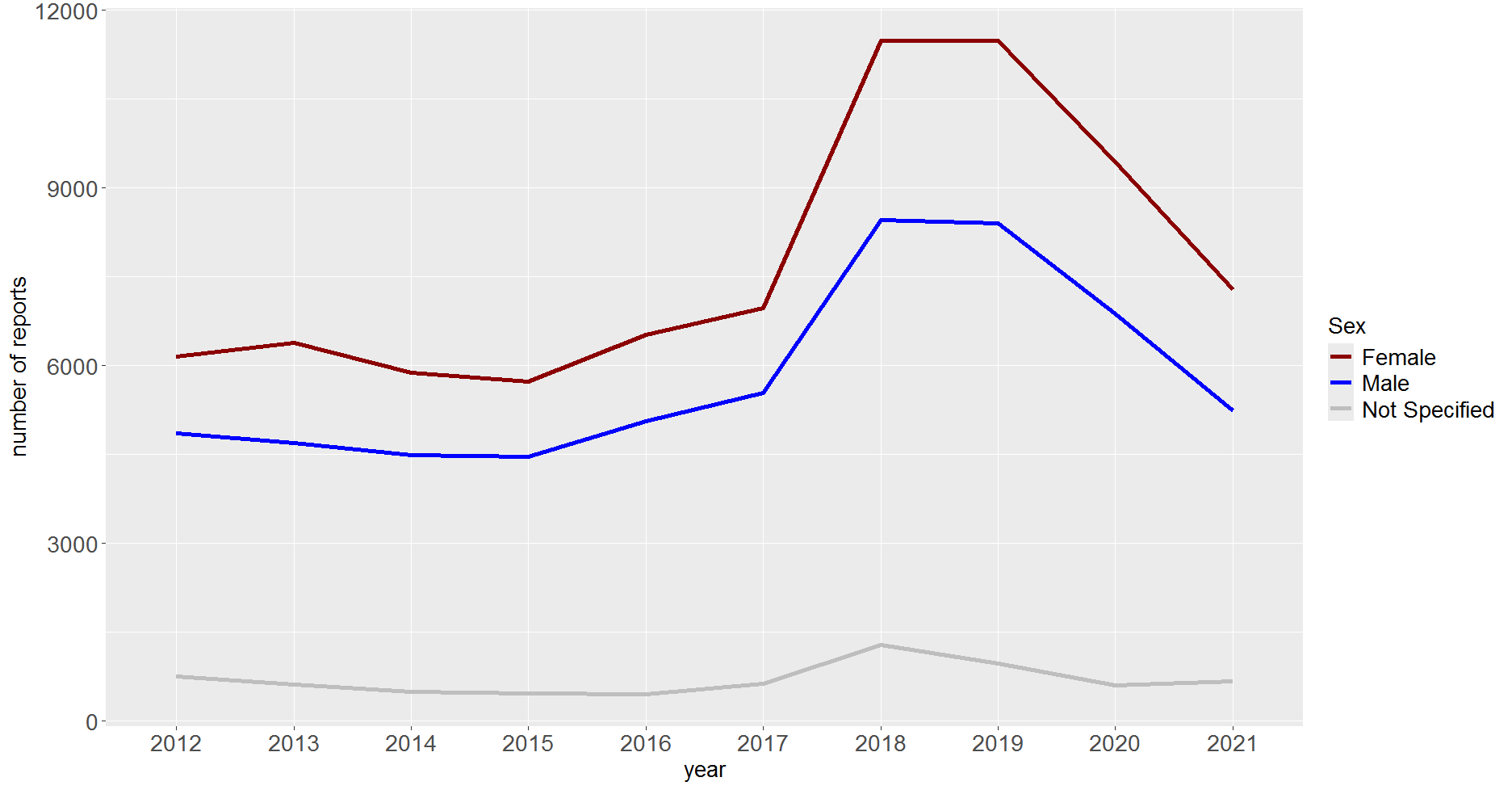


Figure S22 shows the raw number of ADR reports from physicians referring to females, males and unknown sex per year.

Figure S23) Number of ADR reports from consumers per age group.


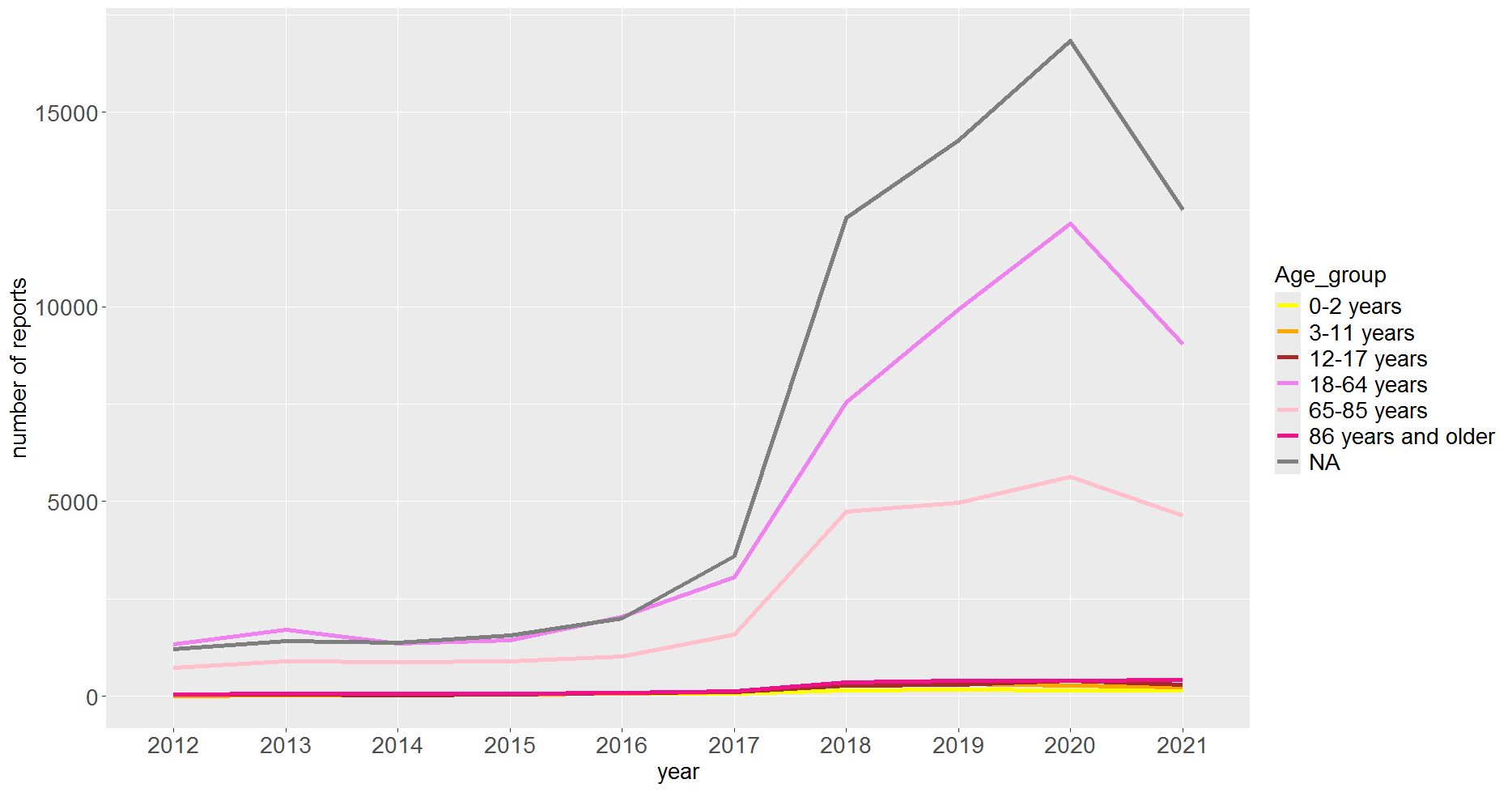


Figure S23 shows the raw number of ADR reports from consumers referring to specific age groups per year.

Figure S24) Number of ADR reports from physicians per age group.


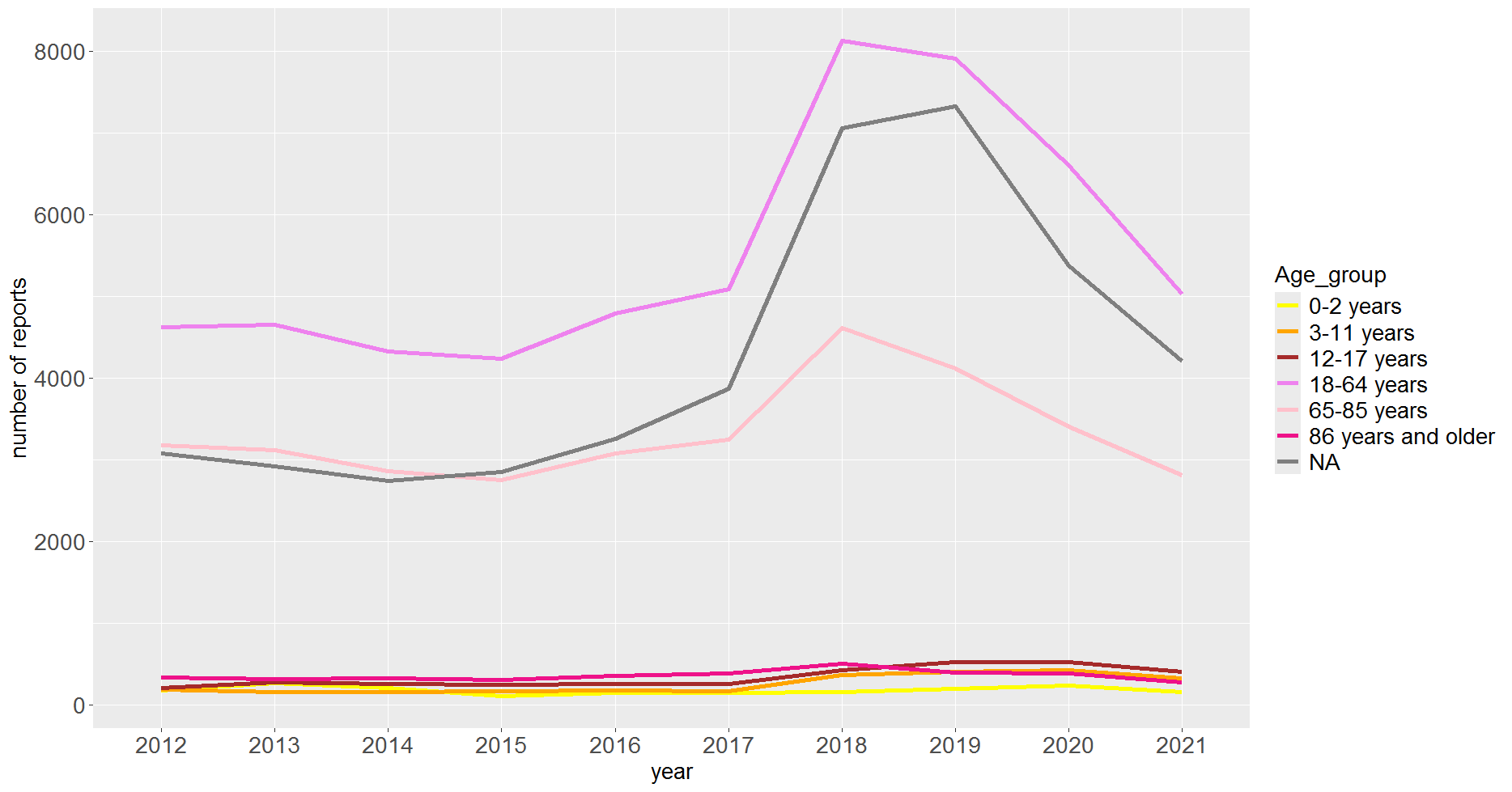


Figure S24 shows the raw number of ADR reports from physicians referring to specific age groups per year.

Figure S25) Reporting rates of ADR reports from physicians and consumers referring to patients aged 0-2 years per 10,000 patients with outpatient drug prescriptions per year.


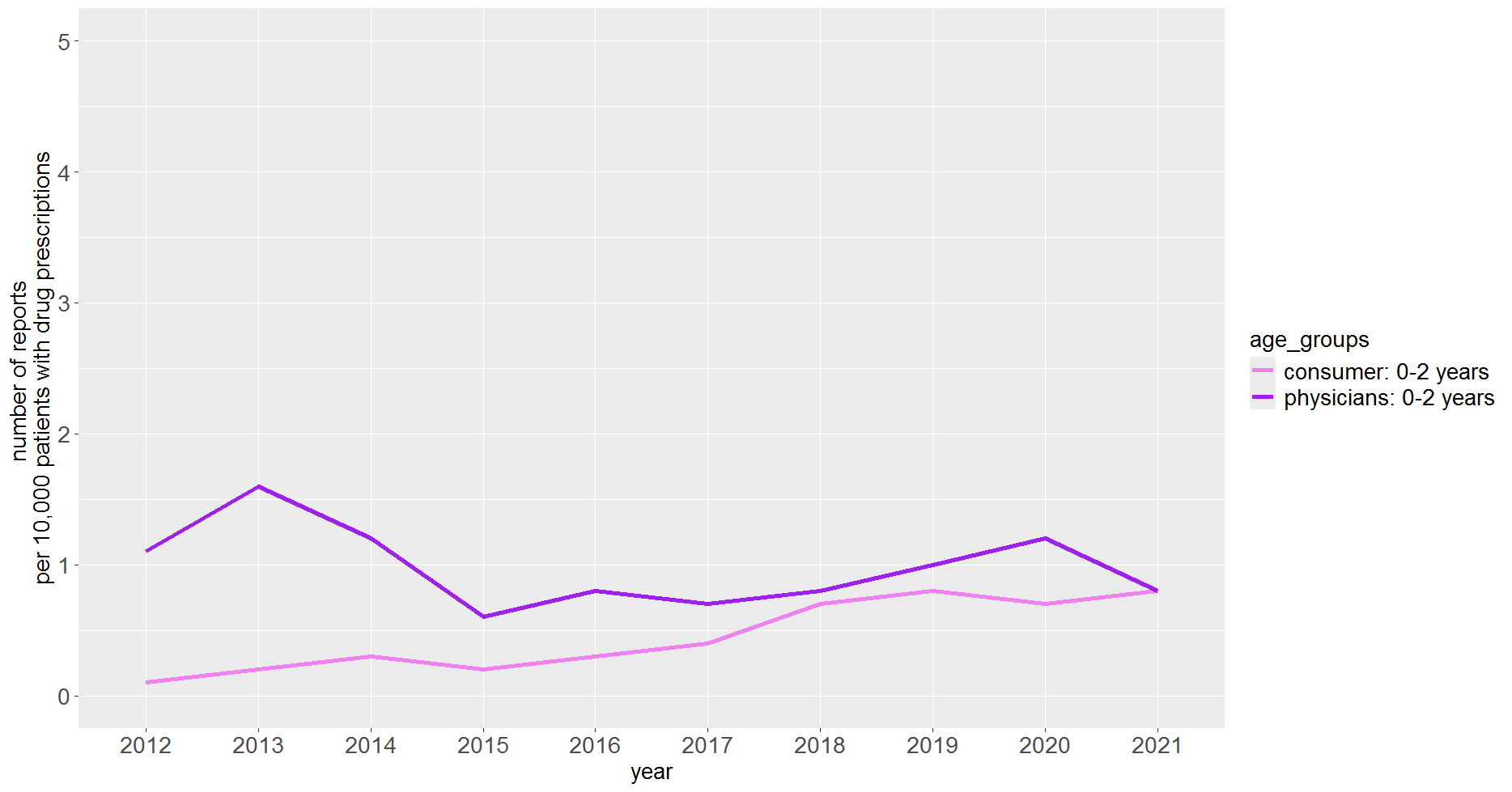


Figure S25 shows the reporting rates of ADR reports from physicians and consumers referring to patients aged 0-2 years.

Figure S26) Reporting rates of ADR reports from physicians and consumers referring to patients aged 3-11 years per 10,000 patients with outpatient drug prescriptions per year.


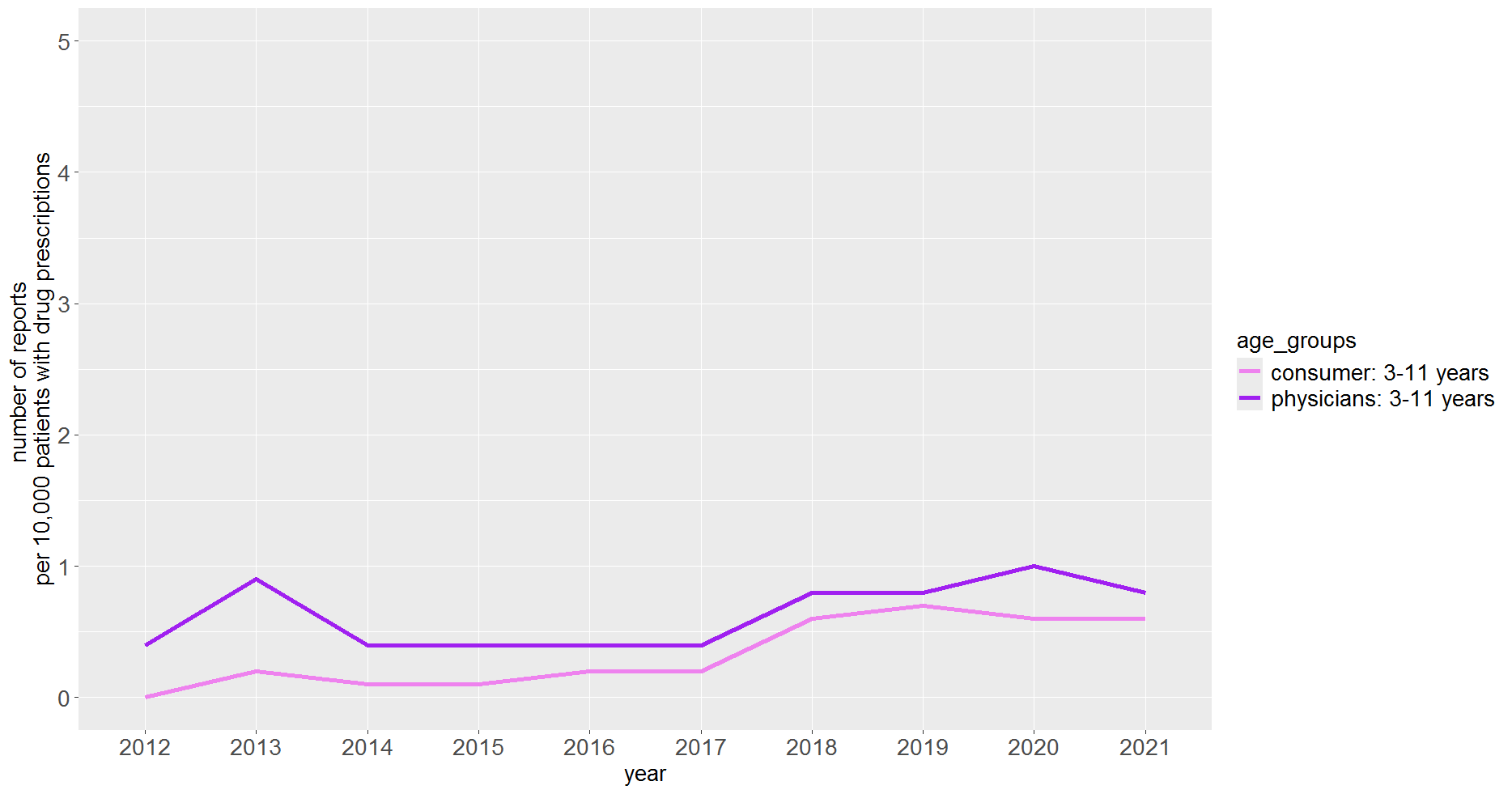


Figure S26 shows the reporting rates of ADR reports from physicians and consumers referring to patients aged 3-11 years.

Figure S27) Reporting rates of ADR reports from physicians and consumers referring to patients aged 12-17 years per 10,000 patients with outpatient drug prescriptions per year.


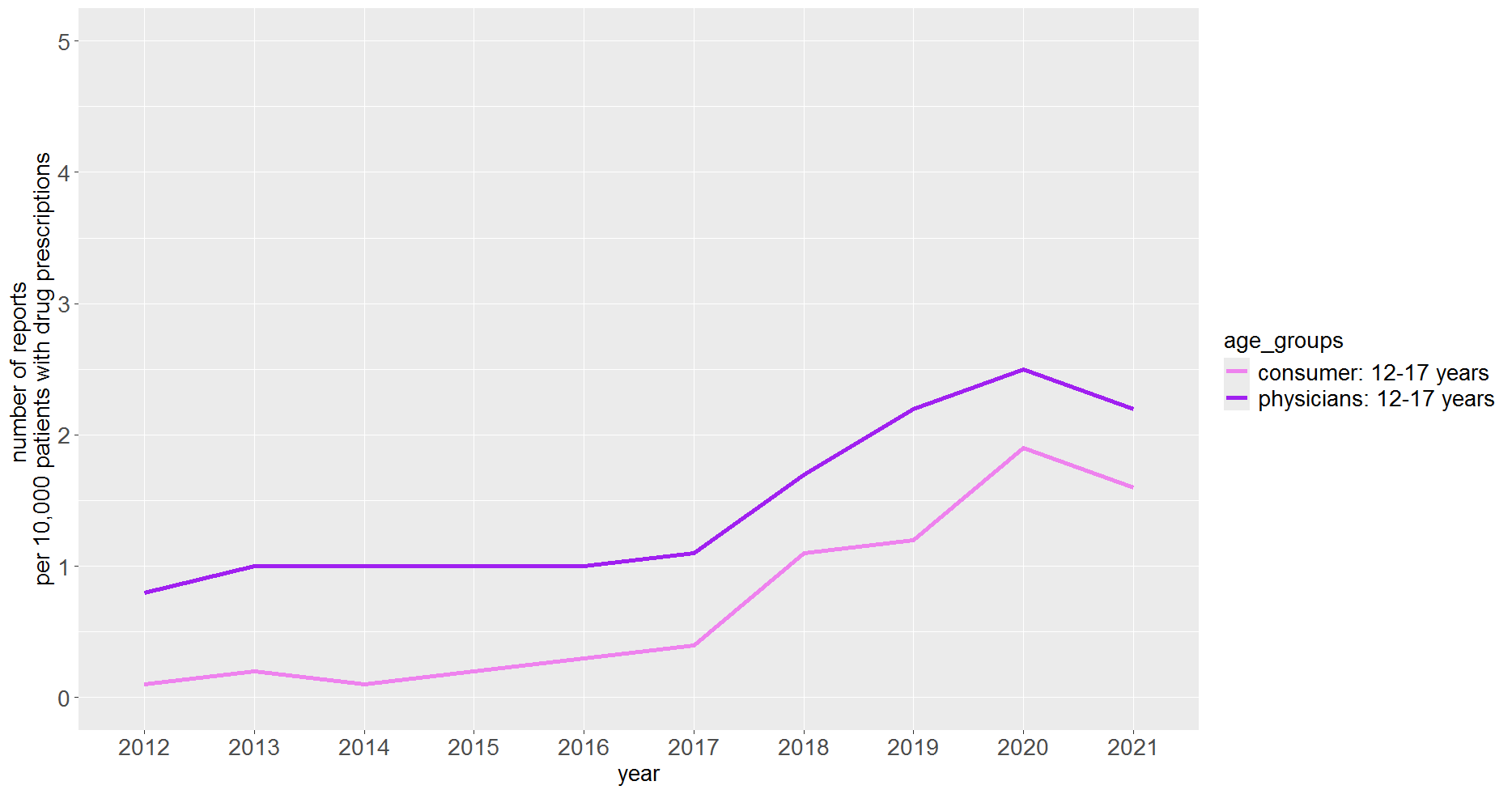


Figure S27 shows the reporting rates of ADR reports from physicians and consumers referring to patients aged 12-17 years.

Figure S28) Reporting rates of ADR reports from physicians and consumers referring to patients aged 18-64 years per 10,000 patients with outpatient drug prescriptions per year.


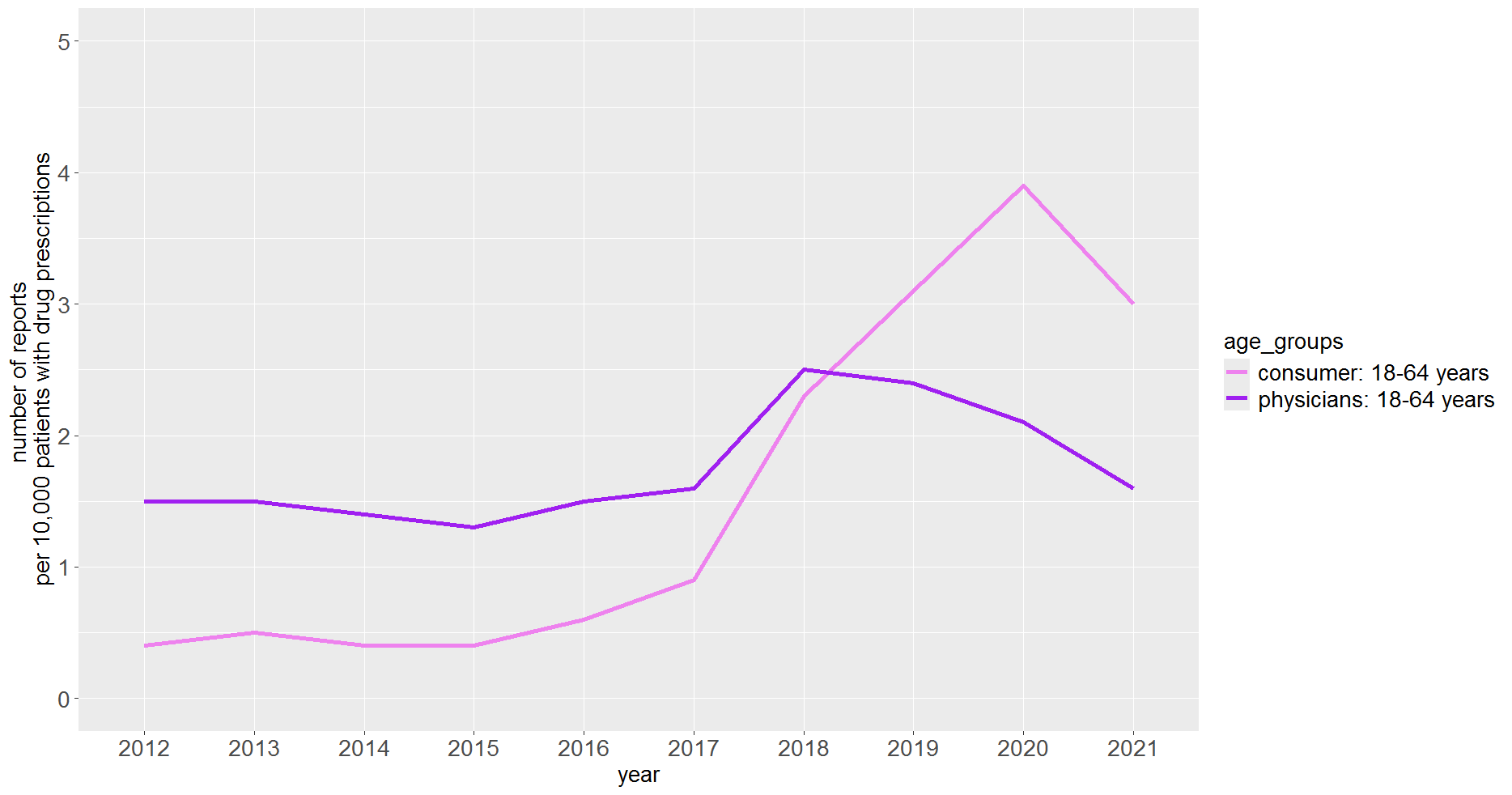


Figure S28 shows the reporting rates of ADR reports from physicians and consumers referring to patients aged 18-64 years.

Figure S29) Reporting rates of ADR reports from physicians and consumers referring to patients aged 65-85 years per 10,000 patients with outpatient drug prescriptions per year.


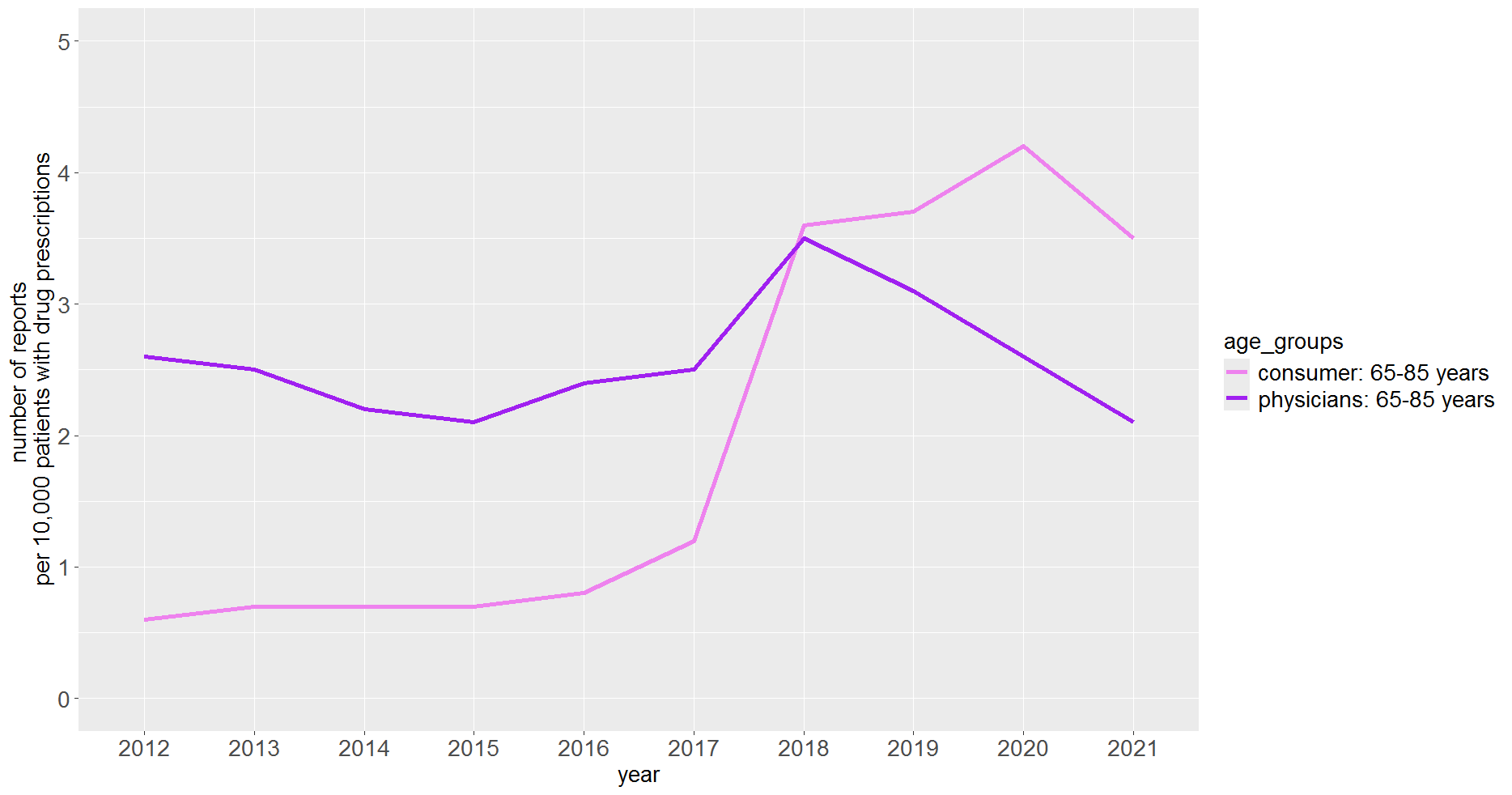


Figure S29 shows the reporting rates of ADR reports from physicians and consumers referring to patients aged 65-85 years.

Figure S30) Reporting rates of ADR reports from physicians and consumers referring to patients aged ≥ 86 years per 10,000 patients with outpatient drug prescriptions per year.


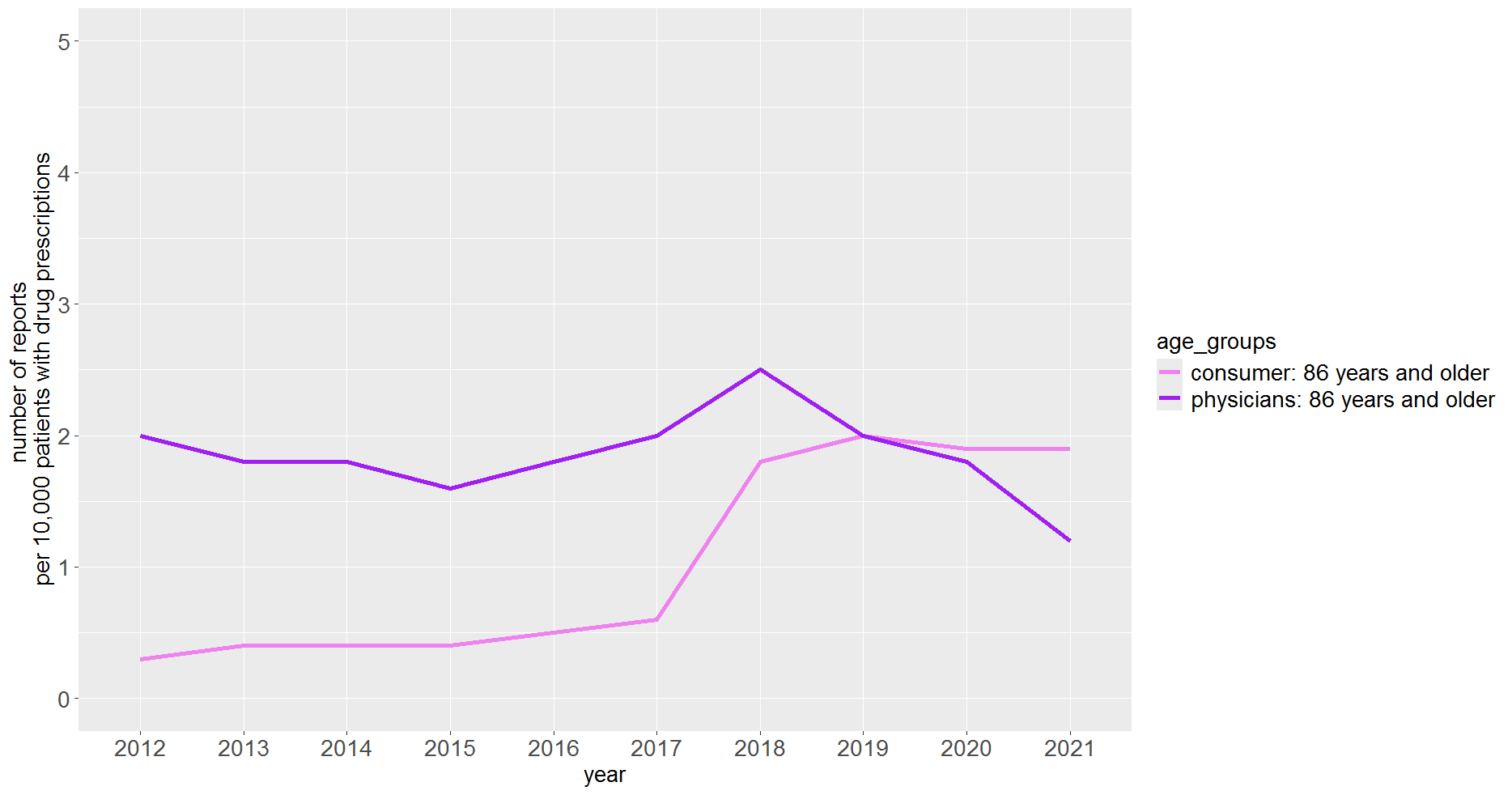


Figure S30 shows the reporting rates of ADR reports from physicians and consumers referring to patients aged ≥ 86 years.
